# *Novabeads*: Stimuli‐Responsive Signal‐Amplifying Hydrogel Microparticles for Enzymeless Fluorescence‐Based Detection of microRNA Biomarkers

**DOI:** 10.1002/smll.202503990

**Published:** 2025-06-25

**Authors:** Haoliang Lu, Fatimah Samman, Erol Hasan, Dana Alsulaiman

**Affiliations:** ^1^ Division of Biological and Environmental Science and Engineering King Abdullah University of Science and Technology (KAUST) Thuwal 23955‐6900 Saudi Arabia; ^2^ Material Science and Engineering Program Division of Physical Science and Engineering King Abdullah University of Science and Technology (KAUST) Thuwal 23955‐6900 Saudi Arabia

**Keywords:** biosensing, microRNA, peptide nucleic acid, smart hydrogel, stimuli‐responsive

## Abstract

Robust and ultrasensitive biosensing platforms for detecting clinically relevant biomarkers from liquid biopsies are vital for precision diagnostics. However, detecting low‐abundance biomarkers such as microRNA typically necessitates complex and costly enzyme‐based strategies like PCR or isothermal amplification. Here, a materials‐driven approach is leveraged to rationally design stimuli‐responsive, signal‐amplifying, and graphically‐encoded hydrogel microparticles, termed Novabeads, for enzyme‐free and fluorescence‐based biomarker detection. Novabeads incorporate pH‐responsive acrylic acid moieties within a polyethylene glycol diacrylate‐based network, enabling significant volume reduction (≈5 fold) upon pH modulation. This stimuli‐responsive shrinking, coupled with high bioreceptor loading via thiol‐ene click chemistry, enables rapid, enzyme‐free optical signal amplification. As a proof‐of‐concept, fluorescently‐labeled peptide nucleic acid (PNA) probes are designed for detecting the cancer biomarker miR‐16, via a fluorogenic Förster resonance energy transfer (FRET)‐based signal. Novabeads exhibit >30 fold signal enhancement over equivalent conventional hydrogel microparticles, driven by three synergistic mechanisms: increased probe loading (≈2.6 fold), enhanced target capture (≈2.8 fold), and shrinkage‐driven amplification (≈5 fold), ultimately leading to over 7 fold reduction in detection limit (28.8 pM; 2.9 fmol), and an expanded linear dynamic range. This rationally designed materials‐driven biosensing strategy enables next‐generation robust, versatile and enzyme‐free biosensors for liquid biopsy diagnostics.

## Graphical abstract

*Stimuli‐responsive graphically‐encoded hydrogel microparticles, termed Novabeads*, achieve rapid, enzyme‐free, multiplexed detection of biomarkers at picomolar sensitivity (LOD = 28.8 pM). Leveraging pH‐responsive signal amplification and dense bioreceptor immobilization, *Novabeads* yield a 35‐fold fluorescence enhancement over standard hydrogel microparticles. Using cancer biomarker miR‐16 as a proof‐of‐concept target, this materials‐driven technology provides robust, versatile, and enzyme‐free biosensing, paving the way for next‐generation liquid biopsy diagnostics.

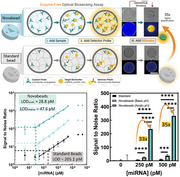

## 1 Introduction

Robust, specific, and ultrasensitive detection of clinically relevant biomarkers from liquid biopsy samples holds transformative potential in minimally‐invasive disease diagnosis, monitoring, and precision healthcare. However, reliable detection of low‐abundance biomarkers like microRNA from biological fluids necessitate the use of complex target or signal amplification strategies to achieve sufficient sensitivity.^[^ ^1^ ^]^ For nucleic acid detection, conventional approaches like polymerase chain reaction (PCR)^[^ ^2^ ^]^ require enzymes, bulky equipment for thermal cycling, and trained personnel.^[^ ^3^, ^4^ ^]^ Advancements in isothermal amplification strategies such as loop‐mediated isothermal amplification,^[^ ^5^ ^]^ exponential amplification reaction,^[^ ^6^ ^]^ strand‐displacement amplification,^[^ ^7^ ^]^ rolling circle amplification (RCA),^[^ ^8^ ^]^ and duplex‐specific nuclease signal amplification^[^ ^9^ ^]^ have considerably advanced biosensing strategies by eliminating the need for thermal cycling. While promising, these methods require complex and costly oligonucleotide probes, enzymes, and/or multi‐step approaches, limiting assay robustness and amenability for point‐of‐care testing.

A conceptually distinct approach toward signal amplification involves the use of materials.^[^ ^10^ ^]^ In optical biosensors, 0D nanomaterials such as quantum dots, iron oxide nanoparticles,^[^ ^11^ ^]^ and gold nanoparticles have been successfully exploited as signal readouts. More recently, smart or stimuli‐responsive materials have been exploited in biosensing applications. Smart materials undergo conformational and chemical changes in response to environmental stimuli such as temperature, pH, light, mechanical forces, or electrical fields.^[^ ^12^, ^13^, ^14^ ^]^ For instance, Wang et al.^[^ ^15^ ^]^ exploited a stimuli‐responsive DNA hydrogel for miRNA detection based on recording the flow‐through distance of a hydrogel film (which expands upon target capture) through a capillary tube, demonstrating the promise of materials‐driven biosensing approaches. While smart materials have been extensively exploited in tissue engineering and drug delivery,^[^ ^16^, ^17^ ^]^ their application in biosensing remains limited; nevertheless, recent advances have demonstrated their potential application in the detection of glucose,^[^ ^18^, ^19^ ^]^ nucleic acid,^[^ ^20^ ^]^ proteins,^[^ ^21^, ^22^ ^]^ and enzymes.^[^ ^23^ ^]^ Among the diverse matrices for biosensing, hydrogels which arewater‐swellable 3D networks of hydrophilic polymer chains,^[^ ^24^ ^]^ have garnered significant attention owing to their solution‐like kinetics, exceptional physicochemical tunability, and porous 3D environments ideal for enhanced probe loading and biomolecular interactions.^[^ ^25^ ^]^ Notably, the Doyle group has pioneered the development of graphically‐encoded hydrogel microparticles offering near‐unlimited multiplexing capacity by patterning them with visually distinguishable shapes or codes.^[^ ^26^ ^]^ However, achieving clinically‐relevant sensitivity in biomarker detection has largely relied upon enzyme‐mediated amplification strategies.^[^ ^27^ ^]^ For instance, Juthani et al,^[^ ^28^ ^]^ exploited peroxidase enzymes like streptavidin‐alkaline phosphatase for colorimetric signal amplification, while Alsulaiman et al.^[^ ^29^ ^]^ utilized polymerases like Phi29 to enable RCA for fluorescence‐based detection. Despite their effectiveness, enzyme‐based methods suffer critical limitations, including degradation, batch‐to‐batch variability, and stringent storage conditions. Natural oligonucleotide probes are also susceptible to degradation under extreme physicochemical conditions, while long assay times hinder their translational potential and user‐friendliness, especially in point‐of‐care and resource‐limited settings.^[^ ^30^ ^]^

**Scheme 1:**
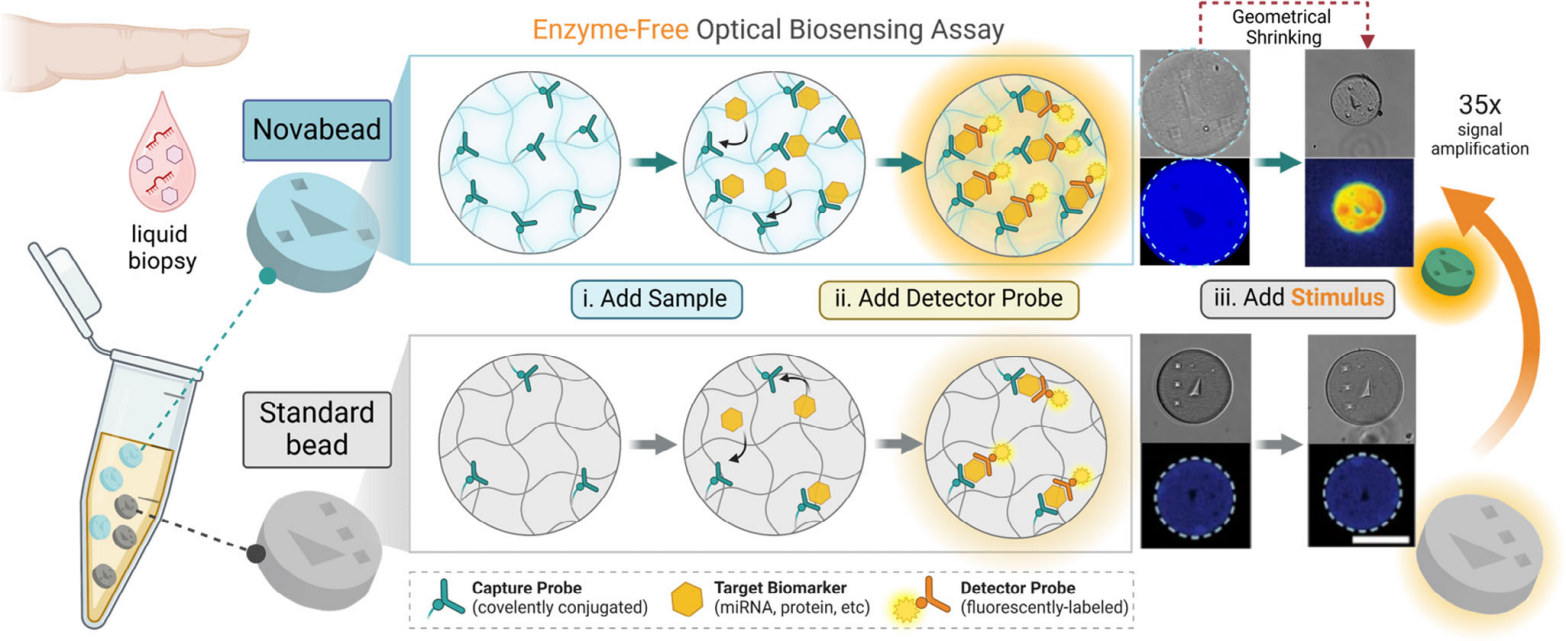
Schematic illustration demonstrating the working principle for enzyme‐free optical biosensing using *Novabead hydrogel microparticles compared to equivalent standard hydrogel microparticles without stimuli‐responsive properties*. Compared to standard beads, *Novabeads* offer greater bioreceptor (capture probe) loading capacity and a geometrical shrinking‐driven signal amplification strategy, enabling over 30 fold signal enhancement in an enzyme‐free manner.

To this end, this work introduces an enzyme‐free materials‐driven strategy enabling isothermal signal amplification within biosensing hydrogel microparticles. Notably, this work represents the first example, to our knowledge, of the rational design of smart materials to achieve enzymeless signal amplification for fluorescence‐based biosensing applications. We report herein the development of the first smart, bioreceptor‐clicked, graphically‐encoded hydrogel microparticles, termed *Novabeads*, for ultrasensitive, enzyme‐free, and multiplexed detection of disease biomarkers (**Scheme** **1**). Compared to equivalent standard hydrogel microparticles, *Novabeads* offer three unique properties: i) a stimuli‐responsive property enabling significant size reduction and instantaneous enzyme‐free optical signal enhancement, and ii) a higher probe loading capacity through a bioorthogonal click conjugation strategy, and iii. synthetic bioreceptors–specifically, peptide nucleic acid (PNA) probes–for robust and specific biomarker detection. All three properties synergistically contribute to *Novabead*’s superior analytical performance and translational potential. As a proof‐of‐concept, we have applied *Novabeads* to the detection of a microRNA biomarker (miR‐16) via a fluorogenic, localized within the microparticles.

## 2 Results

### 2.1 Developing *Novabeads*: Hydrogel Microparticles with Stimuli‐Responsive Properties

**Figure 1:**
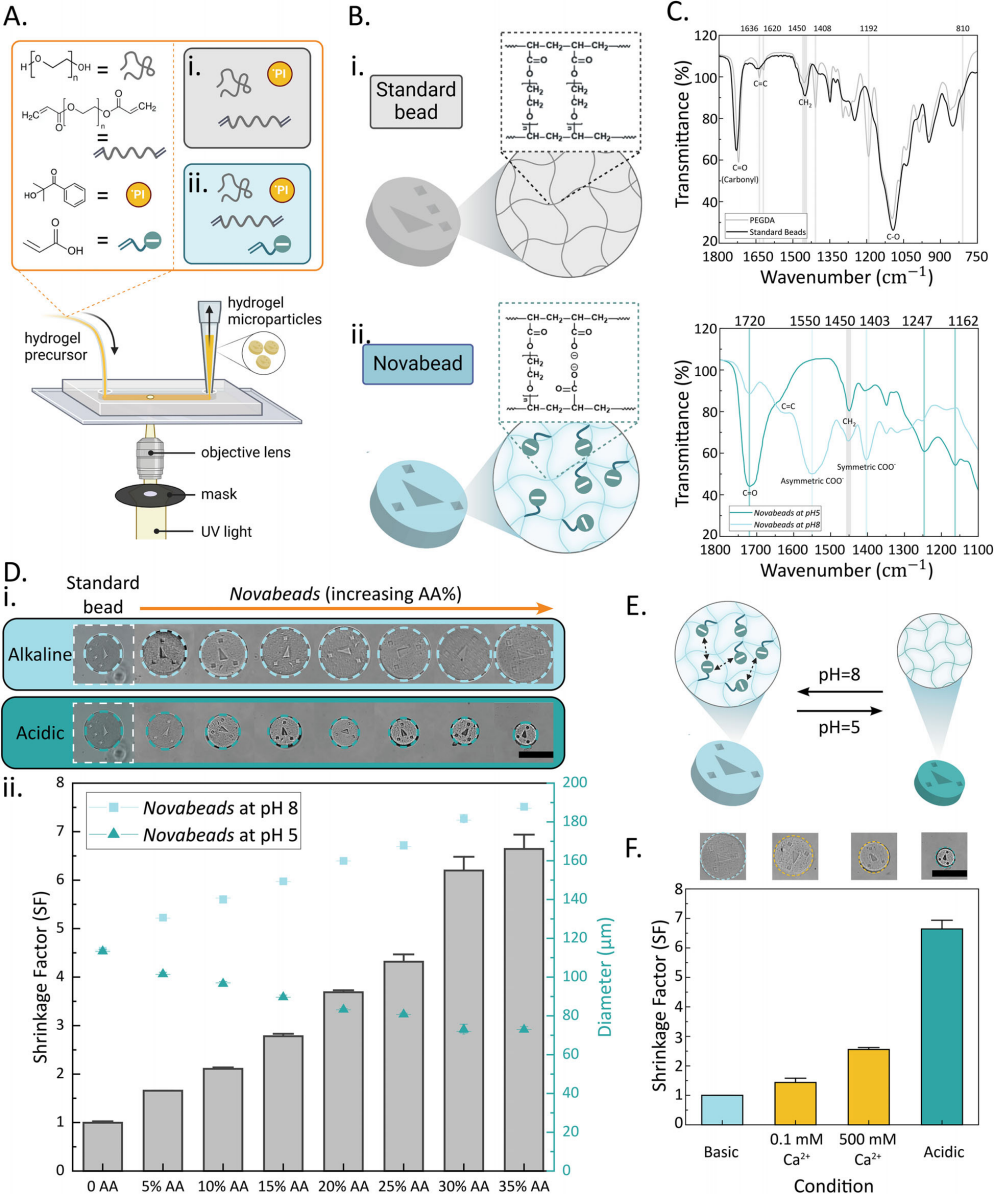
A) Schematic illustration of the stop‐flow‐lithography (SFL)‐based hydrogel microparticle synthesis strategy and composition of the precursor solutions for i) the standard and ii) *Novabead* microparticles. B) Chemical structure of i) standard beads and ii) *Novabeads*. C) (top) FTIR spectra of the standard beads and PEGDA precursor; (bottom) FTIR spectra of the *Novabeads* under acidic and alkaline pH. D) i) Representative brightfield microscopy (BF) images of *Novabeads* with increasing AA concentration under alkaline and acidic conditions (scale bar = 100 µm). The dashed white boxes are used to indicate the same representative standard bead (i.e., control which is not stimuli‐responsive). ii) Diameter of the blank *Novabeads* under alkaline (pH 8, data points in light blue) and acidic (pH 5, data points in dark blue) environments, and the corresponding shrinkage factor (gray bars)(error bars indicate standard deviation). E) Schematic illustration of the pH‐responsive behavior of the *Novabeads*, which can geometrically shrink under acidic pH and swell under alkaline pH. F) Calculated shrinkage factor and corresponding representative BF micrographs of 35% AA *Novabeads* under various stimuli: alkaline conditions, calcium at 0.1 and 500 mM, and acidic conditions.

Fabrication of the standard graphically‐encoded hydrogel microparticles used herein was based on prior work by the Doyle group.^[^ ^31^ ^]^ Briefly, these microparticles, hereafter termed “standard beads”, were prepared by radical photopolymerization of poly(ethylene glycol) diacrylate (PEGDA) through a high‐throughput fabrication strategy called stop‐flow lithography (SFL), which involves selectively exposing a hydrogel precursor solution (monomer stream) within a microfluidic channel to short bursts of UV light through a shape‐defined photomask (**Figure** **1**A).^[^ ^31^ ^]^ UV polymerization results in crosslinking of vinyl groups to create a crosslinked PEGDA network, as shown in Figure 1B. To confirm successful polymerization, Fourier Transform Infrared Spectroscopy (FTIR) analysis was conducted, and the characteristic peaks associated with the acrylate group in PEGDA were monitored (Figure 1C. For the standard beads (i.e., post‐polymerization of PEGDA), these peaks were diminished,^[^ ^32^ ^]^ indicating successful polymerization (Figure 1C).^[^ ^33^, ^34^ ^]^ Specifically, the C═C stretching vibrations from the acrylate group observed at 1636 cm^−1^ and 1620 cm^−1^ were reduced.^[^ ^35^ ^]^ Moreover, the vibrational peaks at 810 cm^−1^ attributed to CH_2_═CH twisting,^[^ ^32^, ^34^ ^]^ 1192 cm^−1^ attributed to acrylic C═O bond,^[^ ^32^, ^34^ ^]^ and 1408 cm^−1^ attributed to CH_2_═CH deformation,^[^ ^32^, ^34^, ^35^ ^]^ were also reduced. The backbone remained intact, as evidenced by minimal changes in its characteristic peaks: the C─O stretch of the ether group at 1094 cm^−1^ and the C─H stretch of the alkane at 2868 cm^−1^ (Figure S1, Supporting Information).^[^ ^35^ ^]^ Additionally, the C═O stretch of the carbonyl group exhibited a slight shift from 1720 cm^−1^ to 1730 cm^−1^ post‐polymerization.^[^ ^33^ ^]^

Regarding our smart *Novabeads*, instead of using enzymes, our hypothesis was to enhance assay sensitivity by concentrating the embedded fluorogenic reporters within a smaller volume and thereby locally amplifying the analytical signal. To achieve this, we sought to endow the standard beads with stimuli‐responsive properties such that an external stimulus can trigger geometrical shrinking of the microparticles. Thus, as presented in the blue box in Figure 1A, to the standard pre‐polymer formulation, we incorporated acrylic acid (AA) moieties to confer the hydrogel network (Figure 1B) with pH‐responsive behavior through protonation and deprotonation of the AA's carboxylic acid functional groups.

To characterize this stimuli responsive behavior, pH 8 was selected as the alkaline environment enabling deprotonation of the carboxyl groups within the hydrogel network while preventing degradation of PNA under extremely high pH conditions.^[^ ^36^ ^]^ On the other hand, pH 5 was chosen as the acidic condition as it approaches the pK_a_ of AA, ensuring protonation of the carboxylic acid groups while avoiding protonation of cytosine and adenine nucleobases under lower pH levels.^[^ ^37^, ^38^ ^]^ The process of protonation and deprotonation influences the FTIR characteristic vibrations of carboxylic acid groups in AA due to conformational changes caused by intermolecular hydrogen bonding between carbonyl groups in response to pH (Figure 1C).^[^ ^39^ ^]^ At pH 5, the carboxyl groups were predominantly protonated, resulting in a pronounced C═O stretching vibration at 1720 cm^−1^ from AA^[^ ^40^ ^]^ and polymerized PEGDA.^[^ ^33^, ^35^ ^]^ A minor peak was also observed at ≈1408 cm^−1^, associated with CH_2_═CH groups of the acrylate from standard beads.^[^ ^32^, ^35^ ^]^ Under alkaline pH 8 conditions, deprotonation occured, leading to the emergence of new peaks at 1550 cm^−1^ and 1403 cm^−1^ attributed to the asymmetrical and symmetrical stretches of the carboxylate ions, respectively.^[^ ^40^, ^41^ ^]^ Additionally, peaks at 1247 cm^−1^ and 1162 cm^−1^, which were present at pH 5, diminish—likely due to the loss of hydrogen bonding between O─H and neighboring C─O groups.^[^ ^42^ ^]^ The small peak at 1720 cm^−1^ remained intact, corresponding to the C═O stretch of the standard beads.^[^ ^33^, ^35^ ^]^ At both pH values, a decrease in the C═C stretch ≈1636 cm^−1^ was observed compared to AA alone (Figure S1, Supporting Information). This suggests that some of these bonds were consumed during the polymerization of AA^[^ ^43^ ^]^ with PEGDA within the hydrogel network. The peak near 1450 cm^−1^, present under both pH conditions, corresponds to the bending vibration of CH_2_ groups from polymerized PEGDA^[^ ^35^ ^]^ and AA^[^ ^40^, ^42^ ^]^ within the polymer network. In *Novabeads*, slight shifts in wavenumber and changes in relative transmittance peak intensities were observed compared to the standard beads. These variations were largely attributed to environmental changes or chemical modifications in the hydrogel network^[^ ^44^ ^]^ following AA incorporation (Figure S2, Supporting Information).

After confirming the chemical structure of *Novabeads* and the presence of the designed responsive functional groups, we sought to study how these changes affect the overall structure and physicochemical properties of the beads in response to the stimulus. To this end, we prepared *Novabeads* with increasing concentrations of AA from 0% to 35%, then measured the resulting hydrogel microparticle diameter under brightfield (BF) microscopy before (pH 8) and after (pH 5) trigger addition. As shown in Figure 1D, increasing the concentration of AA within the hydrogel network resulted in an increase in particle diameter under alkaline conditions, owing to the enhanced electrostatic repulsion between the deprotonated carboxylic acid groups (Figure 1E). Upon switching pH to acidic conditions, the microparticles experienced shrinkage to varying degrees depending on the AA concentration. To characterize the signal enhancement caused by particle shrinkage, the shrinkage factor (SF) was proposed and defined as:

(1) $$\mathrm{Shri}\mathrm{nkage}\;\mathrm{Fac}\mathrm{tor}={\left(\frac{d_{\mathrm{alka}\mathrm{line}}}{d_{\mathrm{acid}\mathrm{ic}}}\right)}^{2}$$

where *d* is the diameter of the microparticles under the respective pH conditions. As shown in Figure 1D, the shrinkage factor of the hydrogel microparticles increased with increasing AA concentration from ≈2 to 6.5 fold for 35% AA. Next, we investigated whether the microparticles could exhibit multi‐responsive behavior. Specifically, we characterized the *Novabeads’* response to other stimuli such as teh addition of divalent (Ca^2+^) or trivalent (Fe^3+^) cations. Interestingly, owing to ionic or electrostatic interactions, the microparticles demonstrated shrinkage with increasing concentrations of both cations, albeit to a much lower extent than that associated with the pH trigger (Figure 1F). It is noteworthy that the ferric ions triggered high shrinkage (up to SF of 6.5) but displayed surface disruption and morphological distortion as shown in Figure S4 (Supporting Information).

### 2.2 Designing PNA Probes for Enhanced Probe Loading Capacity

**Figure 2:**
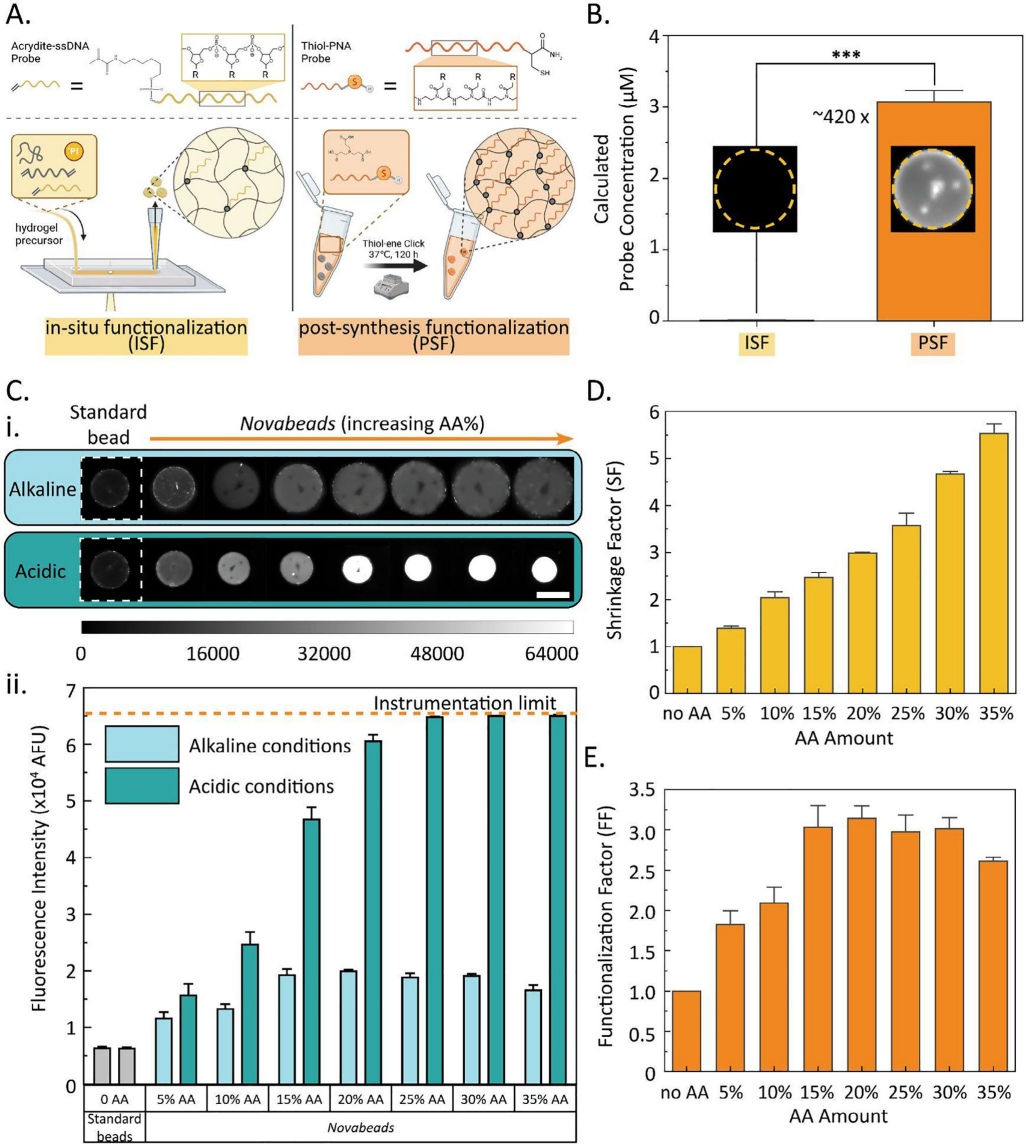
A) Schematic representation of the two probe biofunctionalization strategies: (left) ISF using acrydite‐modified probes and (right) PSF using thiolated probes. B) Quantification of the hydrogel microparticle's loaded probe concentration based on a calibration curve of Cy3 and Cy5 dyes. ^***^ denotes *p*‐value < 0.001 from a *t*‐test. C) Fluorescence microscopy images of hydrogel microparticles after PSF of Cy3‐modified PNA under alkaline and acidic conditions (scale bar shows 100 µm), and ii) Fluorescence micrographs of representative standard and *Novabead* under alkaline (light blue) and acidic (dark blue) conditions following PSF with Cy3‐PNA. D) Shrinkage factor (SF) of *Novabeads* with increasing AA concentrations. E) Functionalization factor (FF) of *Novabeads* after PSF with Cy3‐modified PNA.

With our stimuli‐responsive microparticles developed, we next sought to exploit the *Novabeads* platform for biosensing applications, using microRNA as a proof‐of‐concept biomarker. Instead of conventional bioreceptors based on natural oligonucleotides, we sought herein to use more robust alternatives. As illustrated in **Figure** **2**A, PNAs are synthetic oligonucleotide analogues of DNA or RNA, in which the negatively charged sugar‐phosphate backbone is replaced by a neutral pseudopeptide polyamide backbone composed of N‐(2‐aminoethyl) glycine units.^[^ ^45^ ^]^ Compared to their natural counterparts, PNA probes exhibit higher binding affinity to DNA or RNA complements, greater sequence specificity, more resistance to enzymatic degradation, and increased stability under various conditions.^[^ ^46^, ^47^ ^]^ Additionally, PNA/DNA and PNA/RNA duplexes demonstrate greater thermodynamic stability than their equivalent natural counterparts, making PNA highly attractive for advanced biosensing applications. The PNA probes used in this work were synthesized via our established solid‐phase peptide synthesis protocols based on Fmoc chemistry, as explained in detail in the experimental section. The sequence was designed as a 17‐mer probe complementary to miR‐16, along with the introduction of two aspartic residues at the C terminus to improve the probe solubility and specificity toward the target nucleic acid.^[^ ^48^ ^]^ To enable covalent biofunctionalization within the hydrogel network, a cysteine residue was also incorporated at the probe's C terminus. The chemical structure of the thiolated 17‐mer PNA probe is presented in Figure 2A and Supporting Information Figure S6 (Supporting Information). The thiol functional group facilitates a thiol‐ene click chemistry in a post‐synthesis functionalization (PSF) strategy (Figure 2A), which leverages the abundant unreacted vinyl groups present in the hydrogel microparticles following photopolymerization.^[^ ^49^ ^]^ This strategy, first demonstrated by the Bong group,^[^ ^50^ ^]^ has been shown to provide an 8.3 fold higher probe loading compared to the widely used in situ functionalization (ISF) approach based on acrydite chemistry.^[^ ^49^ ^]^ In the ISF strategy, an acrydite‐modified probe can be directly incorporated within the hydrogel network during the photopolymerization step via reactions with the unreacted vinyl groups within the PEGDA network. Since probe loading capacity is a key determinant of biosensor sensitivity,^[^ ^51^ ^]^ we systematically compared the efficiency of the ISF and PSF strategies using our *Novabead* microparticles. For the PSF approach, a thiolated, Cy3‐labeled 17‐mer PNA probe was synthesized and used to quantify probe loading efficiency. For the ISF approach, an equivalent 5′‐end acrydite‐modified, Cy5‐labeled ssDNA probe was purchased commercially and used as a control.

We evaluated the functionalization efficiency of both methods by monitoring the fluorescence intensity of the *Novabead* microparticles (specifically, 35% AA *Novabeads*) following biofunctionalization via the PSF or ISF approach. Using calibration curves (Figure S5, Supporting Information) for both dyes (Cy3 and Cy5), we calculated the concentration of loaded density as shown in Figure 2B. Notably, with the same initial probe concentration, the PSF approach yielded over 400 times higher probe loading capacity compared to the ISF method; thus, this strategy was adopted for PNA immobilization in all subsequent experiments. PNA functionalization via thiol‐ene click chemistry was confirmed by FTIR analysis based on the appearance of characteristic vibrational peaks of the amide bonds in the PNA backbone as discussed in the Supporting Information (Figure S3).

### 2.3 Stimuli‐Triggered Shrinking Enhances the Biosensing Signal

Following successful probe functionalization of the stimuli‐responsive *Novabeads*, we next sought to validate our materials‐driven strategy for optical signal amplification by assessing the impact of particle shrinkage on fluorescence enhancement. To quantify this effect, Cy3‐tagged PNA was used as a model probe, and the fluorescence intensity of the standard beads was compared to that of the *Novabeads*, prepared with increasing concentrations of AA from 5% to 35%. As shown in Figure 2C, two key observations emerged. First, switching from alkaline to acidic conditions led to a significant fluorescence signal enhancement in the *Novabeads*, which scaled with increasing AA concentration, as expected (i.e., by design). This drastic enhancement even led to signal saturation for *Novabeads* with >20% AA due to the readout instrumentation's limitations (Figure 2Cii). This signal enhancement factor was attributed to the geometric size reduction and increase in effective fluorophore concentration upon particle shrinkage. To quantify this effect, the SF was calculated for the PNA‐functionalized *Novabeads* at each AA concentration and plotted in Figure 2D. A similar trend in SF was observed for the PNA‐functionalized *Novabeads* compared to the unfunctionalized *Novabeads*, though slightly lower values were recorded across all AA concentrations. While the PNA‐modified *Novabeads* experienced shrinkage to the same final size as the unmodified *Novabeads*, their swelling capacity under alkaline conditions was silghtly lower, likely owing to hydrophobic interactions between PNA molecules within the hydrogel network due to the backbone of PNA.^[^ ^52^ ^]^

Beyond the expected fluorescence enhancement upon acid‐triggered shrinkage, another major observation emerged from the data in Figure 2Cii. Interestingly, even under alkaline (i.e., swollen) conditions, *Novabeads* at all AA concentrations displayed higher mean fluorescence signals compared to the standard beads, indicating greater probe immobilization within the *Novabeads* despite identical biofunctionalization conditions. This increase in probe loading was attributed to the *Novabeads*’ larger mesh size under alkaline conditions (i.e., swollen conditions), driven by electrostatic repulsion between AA moieties, enabling more efficient probe incorporation within the hydrogel network. To quantify this enhancement in biofunctionalization efficiency, we introduced the functionalization factor (FF)–defined as the fluorescence intensity ratio of *Novabeads* to standard beads under alkaline conditions:

(2) $$Functionalization\;Factor=\frac{I_{Novabead\_alkaline}}{I_{standard\_alkaline}}$$

The FF was plotted against AA concentration (Figure 2E), revealing an increasing trend reaching a maximum of ≈3 fold enhancement at the 20% AA condition. A slight decline in FF was observed in the 35% AA *Novabeads*, attributed to the reduced effective concentration of reactive vinyl groups under these conditions. These findings reveal that our *Novabeads* offer signal enhancement not only through stimuli‐triggered geometrical shrinkage but also through enhanced probe loading effects.

**Figure 3:**
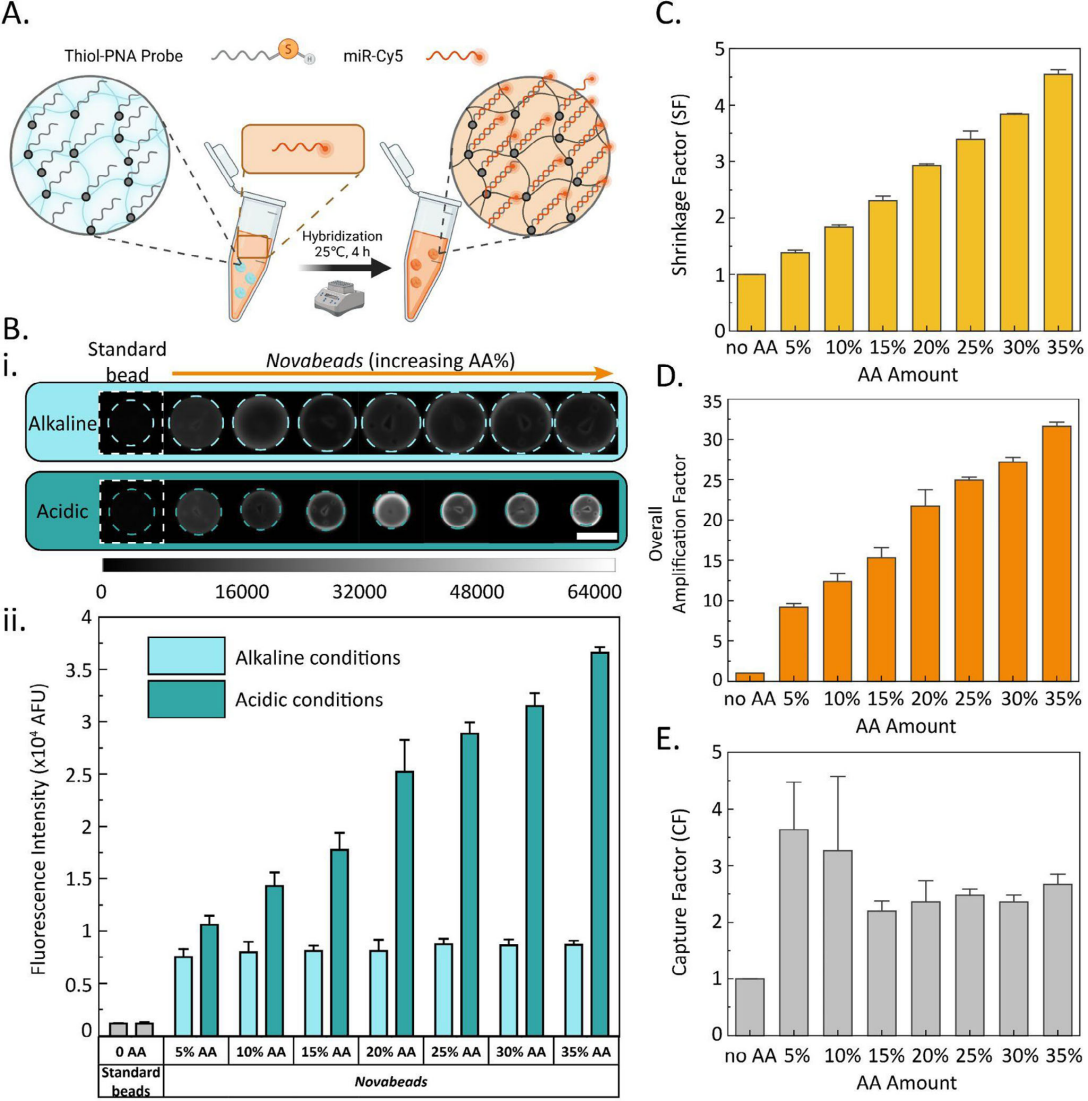
A) Schematic illustration of the experimental setup. B) i) Fluorescence micrographs (i) and average fluorescence intensity of standard beads and *Novabeads* under alkaline and acidic conditions, following hybridization with miR‐16‐Cy5 (scale bar shows 100 µm). C) Shrinkage factor (SF) of *Novabeads* following hybridization with miR‐16‐Cy5. Overall amplification factor (D) and capture factor (E) of *Novabead* microparticles with increasing AA concentrations.

To confirm this hypothesis and further assess the signal‐amplifying capacity of our *Novabeads*, we evaluated their response using fluorescently‐labelled target miRNA (**Figure** **3**A). In this case, the standard beads and *Novabeads* (at all AA concentrations) were functionalized with identical initial PNA probe concentrations (1 µM), without fluorescent labelling. Following a 4 h incubation with 100 nM of target Cy5‐tagged miRNA, all beads were washed thrice, then imaged with a fluorescence microscope (see Experimental Section). The fluorescence signal intensities of all beads were then quantified under both alkaline (swollen) and acidic (shrunk) conditions, and plotted in Figure 3Bii.

Consistent with our previous results, the *Novabeads* exhibited significantly higher fluorescence signals compared to the standard beads (Figure 3Bii). To elucidate this amplification mechanism, the SF of the *Novabeads* was first calculated and plotted in Figure 3C, revealing a similar trend to the blank and PNA‐functionalized *Novabeads* but with a lower maximum SF of 4.5 fold at 35% AA. This difference can be attributed to the electrostatic repulsion between miRNA strands within the hydrogel network, which may limit particle shrinkage. To quantify overall signal enhancement, we introduced the overall amplification factor, defined as the fluorescence intensity ratio of *Novabeads* under acidic conditions (i.e., post‐trigger) to the standard beads under alkaline conditions:

(3) $$\mathrm{Over}\mathrm{all}\;\mathrm{Ampli}\mathrm{fica}\mathrm{tion}\;\mathrm{Fact}\mathrm{or}=\frac{I_{\mathrm{Nova}\mathrm{bead}\_\mathrm{acid}\mathrm{ic}}}{I_{\mathrm{Stan}\mathrm{dard}\_\mathrm{alka}\mathrm{line}}}$$

The overall amplification factor as a function of AA concentration was plotted in Figure 3D, revealing unexpectedly high values, with the 35% AA *Novabeads* showing over 30 fold signal amplification. Importantly, the product of FF and SF alone could not fully account for the observed enhancement, as summarized in **Table** **1**. For example, for the optimal 35% AA *Novabeads*, FF (2.6) and SF (4.6) would predict only a 12‐fold signal enhancement compared to the standard beads—significantly lower than the observed amplification factor. This ≈3 fold discrepancy suggests the presence of an additional, significant, and previously unaccounted‐for amplification mechanism within our *Novabeads* occurring during hybridization or target capture. To quantify this effect, we introduced the capture factor (CF), defined as follows:

(4) $$Capture\;Factor\;\left(CF\right)=\frac{I_{Novabead\_alkaline}}{I_{standard\_alkaline}\times FF}$$

**Table 1:** Summary of all the amplification factors from smart microparticles for biosensing applications, along with their product value compared to the overall amplification. The numbers for each factor represent the average value extracted from the plots.

		*Novabeads* [% AA]						
	Standard beads	5%	10%	15%	20%	25%	30%	35%
FF	1	1.8	2.1	3.0	3.1	3.0	3.0	2.6
SF	1	1.4	1.8	2.3	2.9	3.4	3.8	4.6
CF	1	3.6	3.3	2.3	2.3	2.6	2.4	2.8
Calculated overall amplification factor =*FF* **✗** *SF* **✗** *CF*	1	9.1	12.9	16.0	20.9	25.8	28.2	33.1
Measured overall amplification factor	1	9.2	12.5	15.3	21.8	24.9	27.2	31.6
Percent Difference between calculated and measured amplification factors	NA	0.9%	3.2%	4.5%	4.2%	3.6%	3.7%	4.7%

The CF was plotted for all AA concentrations (Figure 3E), revealing a consistent ≈2.5 fold signal enhancement in *Novabeads* compared to standard beads, irrespective of AA concentration. Interestingly, this enhancement factor did not increase with AA content, likely due to electrostatic repulsion between target miRNA and negatively charged carboxyl groups at higher AA concentrations. This enhancement was at least partially attributed to the higher probe loading within *Novabeads* (as confirmed by the FF), improving target capture efficiency.

To analyze the contributions of each of the three enhancement factors on the overall amplification factor, the FF, SF, and CF for each AA concentration were summarized in Table 1. The product of these three factors was then compared to the measured overall amplification factor, showing less than 5% deviation between the measured and observed overall amplification factors. This agreement confirms that our optimized *Novabead* platform can achieve oevr 30‐fold signal amplification not only through geometrical shrinkage, but via the synergistic interplay of three independent mechanisms–characterized by FF, SF, and CF–amounting to a triple‐enhancement benefit.

### 2.4 *Novabead* Optimization by tuning Damkohler Number

**Figure 4:**
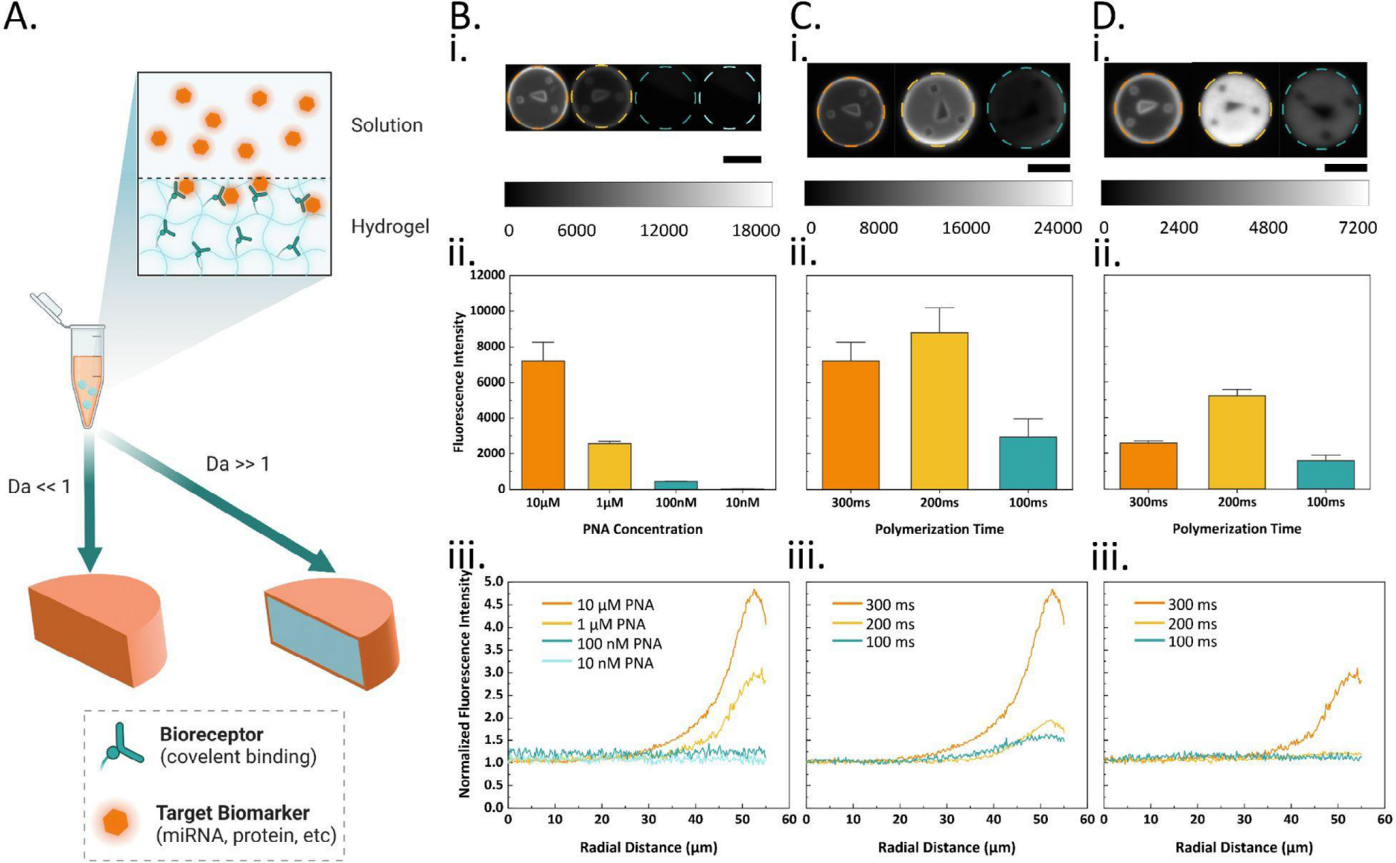
A) Schematic representation of hydrogel microparticles with high or low Da. At high Da (Da >>1), the fluorescence signal becomes localized at the periphery of the microparticle, while a lower Da (Da<< 1) enables a more uniform signal distribution throughout the microparticle. B) Investigating the effect of varying initial PNA probe concentration (10 µM, 1 µM, 100 nM, and 10 nM) on Da. C) Investigating the effect of varying UV photopolymerization time (using 10 µM initial PNA probe concentration) on Da. D) Investigating the effect of varying UV photopolymerization time (using 1 µM initial PNA probe concentration) on Da, showing (i) fluorescence micrographs of representative *Novabeads* (scale bar shows 100 µm), ii) the associated average fluorescence intensity of *Novabeads* and (iii) the fluorescence intensity profile showing signal uniformity of a representative *Novabead*, under the given conditions.

While the triple‐enhancing *Novabeads* demonstrated exceptional signal amplification, a notable drawback was the localization of fluorescence at the microparticle periphery, resulting in a ring‐shaped pattern in the micrographs of all *Novabeads* with an AA concentration above 10% (Figure 3Bi). This effect, previously reported in hydrogel microparticles, suggests that under high AA conditions, the rate of hybridization between the target and probe significantly exceeds the diffusion rate of the target into the hydrogel matrix (**Figure** **4**A).^[^ ^53^, ^54^ ^]^ This relationship can be described by the Damköhler number (Da), a dimensionless parameter that quantifies the ratio of the reaction rate to the diffusive transport rate within a system, as defined in Equation (5) below:

(5) $$Da=\frac{k_{a}P_{0}L^{2}}{D_{gel}}$$

where *k_a_* represents the forward rate constant, *P*_0_ represents the incorporated probe concentration (PNA concentration within the hydrogel), *L* is the particle radius, and *D_gel_* is the diffusivity of the target biomolecule within the hydrogel matrix. Interestingly, the observed boundary layer gets more pronounced as the AA concentration increases, suggesting that these parameters create high Da conditions (Da>>1). Thus, to achieve a more uniform signal distribution without compromising biosensing performance, we optimized the *Novabead* conditions by tuning both *P*_0_ and *D_gel_*. First, to optimize *P*_0_, we varied the initial probe concentration during the thiol‐ene biofunctionalization step of the 35% AA *Novabeads* from 10 nM to 10 µM. After probe functionalization, the *Novabeads* were thoroughly washed and hybridized with fluorescently labeled miRNA (miR‐Cy5, 100 nM) for 4 h under stirring. After a final wash, the microparticles were imaged using a Cy5 filter set to quantify both average signal intensity and the profile of fluorescence intensity across the microparticle. To visualize the signal distribution across particles, intensity profiles were normalized to the fluorescence intensity at the particle center, facilitating direct comparison between conditions. As shown in Figure 4B, lowering the initial PNA probe concentration led to a more homogeneous signal distribution (indicated by a straighter normalized intensity profile), but at the cost of lower overall signal intensity (indicated by the lower mean fluorescence intensity in the bar charts).

Second, to optimize *D_gel_* and characterize its influence on Da, we varied the UV irradiation time of the *Novabeads* during the SFL‐based fabrication step from the original 300 ms down to 100 ms. As UV exposure time affects crosslinking density—and thus the diffusivity of probes within the hydrogel network—this parameter was systematically evaluated while maintaining a constant initial PNA probe concentration of either 10 µM (Figure 4C) or 1 µM (Figure 4D). We then plotted the overall fluorescence intensity (Fiugre 4B, panel ii) and analyzed the corresponding intensity profile of representative microparticles under each condition (Figure 4b, panel iii).

A consistent trend was observed for both PNA concentration conditions, with optimal signal intensity and uniformity achieved at 200 ms UV irradiation. As expected, the highest exposure time (300 ms) resulted in a pronounced ring effect and the lowest uniformity in signal distribution (Figure 4C,D). On the other hand, the lowest exposure time (100 ms) produced the most uniform fluorescence distribution but at the cost of reduced overall signal intensity, likely owing to lower probe incorporation. Considering these findings, the optimal conditions to balance signal distribution and signal intensity in our *Novabead* platform were identified as 200 ms UV polymerization time and 1 µM initial probe concentration via the PSF thiol‐ene click strategy. It is noteworthy that our system is amenable to even higher probe loading capacity, which can be optimized based on specific applications and biosensing requirements.

### 2.5 Implementing the Optimized *Novabeads* in microRNA Biosensing: FRET‐Based Assay

#### 2.5.1 Designing a Fluorogenic FRET‐Based Biosensing Strategy in Hydrogel Microparticles

With the optimized *Novabead* platform, we next sought to validate its enzymeless signal amplifying capacity with a clinically‐relevant circulating biomarker. As proof‐of‐concept, we chose miR‐16, a microRNA whose levels are dysregulated in many cancer types, including cervical, breast, gastrointestinal, and lung malignancies.^[^ ^55^ ^]^ Oligonucleotide‐templated reaction is a powerful and versatile approach that facilitates a range of chemistries through an oligonucleotide template, by bringing reactants into close proximity to achieve sufficient local molarity for reaction initiation. It has been implemented in biosensing by designing a fluorescence‐based readout upon target capture though FRET, QUAL, and QUAL‐FRET assays.^[^ ^56^, ^57^ ^]^

**Figure 5:**
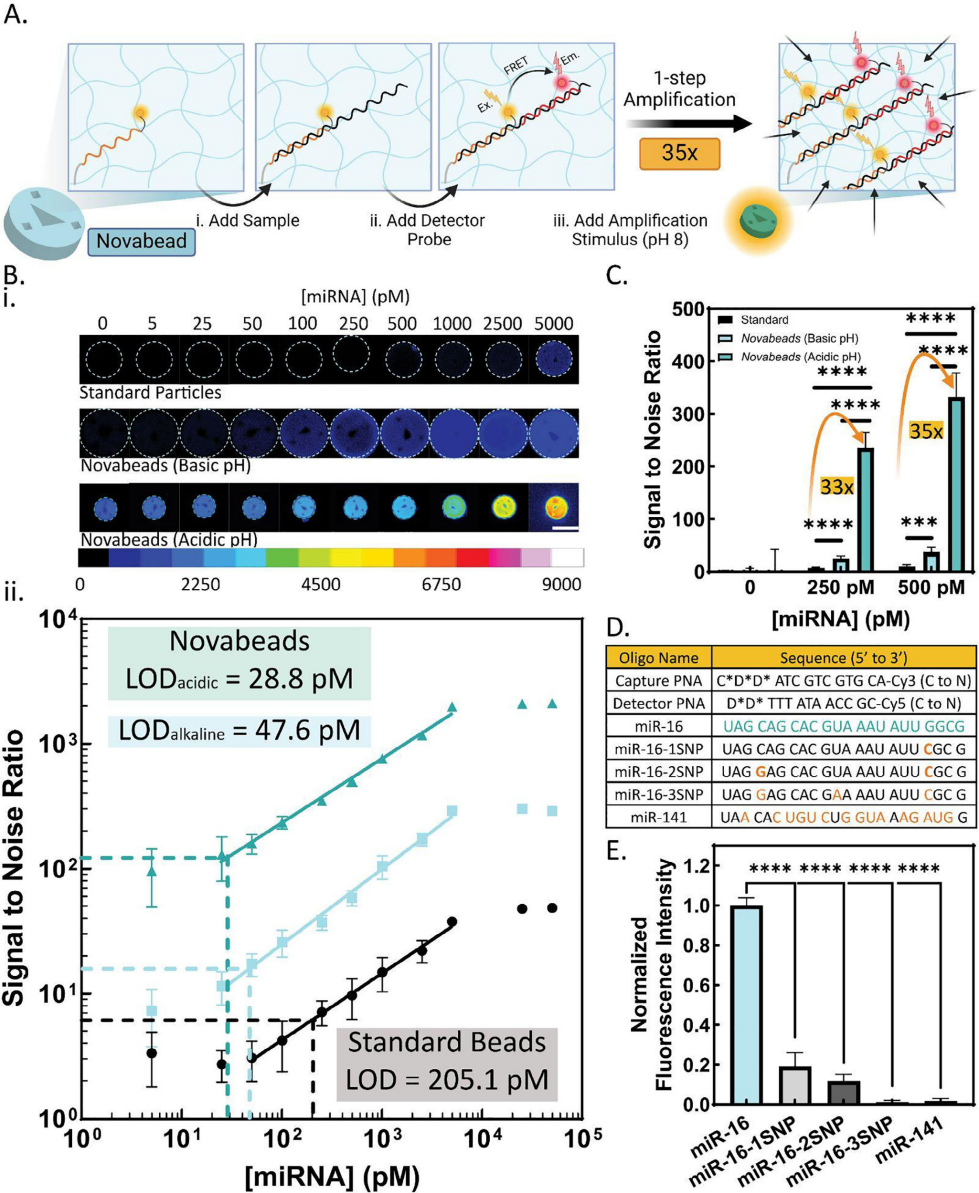
A) Schematic illustration of the biosensing strategy relying upon FRET‐based OTR within encoded *Novabeads* for microRNA detection, involving three steps: i) Adding the biofluid containing miRNA enabling its hybridization to the conjugated capture probe, ii) Adding the detector probe (fluorescently‐labelled) and enabling hybridization and FRET signal generation, and iii) Adding the amplification stimulus (pH 5 buffer) to shrink the *Novabeads* to enable fluorescence signal enhancement. B) Comparison of the biosensing performance of standard and *Novabead* microparticles (under alkaline and acidic conditions) via FRET‐based detection of miR‐16, showing i) Micrographs of representative microparticles (scale bar = 100 µm). ii) Calibration curves of SNR versus miRNA concentration. SNR is calculated as the ratio of the net signal (control‐subtracted signal) to the negative control; the control signal is from the 0 pM condition. Dashed lines indicate LOD as three times the standard deviation of the control (0 pM) condition. C) Comparison of the SNR of the *Novabeads* compared to the standard beads for two selected miRNA concentrations (250 and 500pM). D) Table of oligonucleotide sequences used in this experiment, including the synthesized PNA probes and commercial synthetic miRNAs. Letters in orange indicate the mismatched nucleotide. C^*^ = cysteine residue. D^*^ = aspartic acid residue. E) Specificity test for the optimized *Novabeads* (35% AA) under acidic conditions, demonstrating the FRET signal response to target miR‐16 (normalized to 1, light blue) compared with non‐target miR‐141 and three mutated miR‐16 sequences with one, two, and three individual SNPs. ^****^ denotes *p*‐value < 0.0001 (*t*‐test).

Herein, the system was designed such that the presence of the target miRNA brings a FRET pair of dyes into close proximity (within 1–10 nm),^[^ ^58^ ^]^ thereby generating a specific and quantitative signal. Specifically, two PNA probes labeled with either a Cy3 (donor) or Cy5 (acceptor) dye were designed to be complementary to the 3′ or 5′ end of miR‐16, such that hybridization to the target enables a non‐radiative energy transfer bteween the two dyes and a corresponding FRET signal generation (**Figure** **5**A).^[^ ^59^ ^]^ To incorporate this strategy within our optimized *Novabead* platform (35% AA *Novabeads*), the Cy3‐labelled PNA (termed capture probe; Figure 5A orange probe) was conjugated into the hydrogel based on the optimized PSF biofunctionalization strategy previously described. The second Cy5‐labelled PNA (termed detector probe; Figure 5A pink probe) was kept in solution. The precise positioning of the dyes along the PNA probes was carefully designed to ensure that the presence of target miRNA enables the formation of a templated hybridization complex, bringing the FRET pair within optimal proximity (≈11 nt apart) for efficient energy transfer (Figure 5A).

With this design, the biosensing strategy involved three steps: i) addition of the biofluid sample (containing miRNA) to enable hybridization, for 4 h under stirring and room temperature (RT) conditions, ii) introduction of the detector PNA, which was incubated for 4 h under stirring and RT conditions, and iii) addition of the stimulus (i.e., exposure to pH 5 buffer) to trigger signal amplification. Following each step, the microparticles were rinsed three times with rinse buffer (RB) to remove non‐specifically bound molecules. Finally, at least five microparticles from each assay condition were imaged using brightfield and fluorescence microscopy with a filter set optimized for the FRET pair. Image analysis was conducted using ImageJ software, where a region of interest (ROI) was defined around each microparticle based on brightfield micrographs to quantify fluorescence intensity.

#### 2.5.2 Characterization of *Novabeads*’ Analytical Performance: Sensitivity and Specificity

With a proof‐of‐concept optical biosensing strategy developed, we next characterized the analytical performance of the *Novabeads* in comparison to standard beads. First, the platform sensitivity was assessed by generating calibration curves, where target miRNA concentrations were varied from 5 pM to 50 nM in a 100 µL assay volume, using the same biosensing approach with either standard beads or *Novabeads* (under alkaline and acidic conditions). Figure 5Bi shows false‐colored fluorescence micrographs of representative microparticles following the FRET‐based biosensing assay completion, for the standard beads (top) and *Novabeads* (bottom), before and after the stimulus (pH trigger). Notably, when plotted using the same calibration color bar, the micrographs reveal a drastic signal enhancement using the *Novabead* platform. To quantify this performance, signal intensities were normalized and plotted as signal‐to‐noise ratio (SNR), calculated as the ratio of the control‐subtracted signal to the control signal (at 0 pM miRNA). The resulting SNR plots across miRNA concentrations for each system (Figure 5Bii), confirmed the *Novabead* platform's superior sensitivity, dynamic range, and overall SNR.

Linear regression analysis was applied to the linear regions of each calibration curve in Figure 5B, yielding the following equations: *y*  =  0.53*x* − 0.44 (*R* ^2^ =  0.996), *y*  =  0.60*x* + 0.19 (*R* ^2^ =  0.994), and *y*  =  0.51x + 1.35 (*R* ^2^ =  0.994) for the standard beads, *Novabeads* in alkaline, and *Novabeads* in acidic conditions, respectively, where y represents the logarithm of the SNR value, and x represents the logarithm of the miRNA concentration. The limit of detection (LOD), defined as three times the standard deviation of the control condition, was calculated for each microparticle system. Compared to the standard beads with an LOD of 205.1 pM, *Novabeads* exhibited an LOD of 47.6 pM and 28.8 pM under alkaline conditions (i.e., before pH trigger) and acidic conditions (i.e., after pH trigger), respectively. Moreover, the linear dynamic range also expanded by one order of magnitude from 100–5000 pM for the standard beads to 25–5000 pM for the *Novabeads* (after pH trigger). Compared to previously reported enzyme‐free biosensing strategies, our enzymeless *Novabead* platform achieved a competitive LOD and broad dynamic range (Table S1, Supporting Information). While some alternative strategies may offer comparable or superior sensitivity, they often rely on nanomaterials that present challenges, including stability, high complexity, and limited scalability, hindering clinical translation.^[^ ^60^ ^]^ In contrast, the *Novabead* platform offers a simpler, more stable, and scalable alternative that achieves high‐sensitivity detection without the complexities of nanomaterials or enzyme‐based amplification, making it more suitable for practical and translational applications.

Beyond lowering the detection limit, *Novabeads*–under alkaline and even more so under acidic conditions–exhibited significantly higher SNR at all miRNA concentrations compared to standard beads. To quantify the degree of SNR enhancement, three target miRNA concentrations (250, 500, and 1000 pM) were selected, and the corresponding SNR values following their detection were plotted in Figure 5C. The *Novabeads* (post‐trigger) exhibited a 33‐fold and 35‐fold enhancement in SNR for the 250 pM and 500 pM miRNA conditions, respectively, relative to standard beads. Importantly, these amplification factors matched the theoretical and expected amplification factors based on the triple‐enhancement effect outlined in Table 1. Interestingly, an even more drastic improvement in SNR was found when comparing our Novabead hydrogel‐based assay to equivalent solution‐based assays, as highlighted in the Supporting Information (Figure S13, Supporting Information). Notably, a ≈106‐fold enhancement in SNR was observed for the detection of 100 nM miRNA (under alkaline conditions, even without pH‐triggered geometrical amplification) compared to equivalent solution‐based assays. This significant enhancement factor offered by the *Novabead* platform opens the possibility for detecting rare or previously undetectable biomolecular targets using smaller and more cost‐effective optical detection platforms, eliminating the need for high‐end microscopy setups or enzyme‐based amplification strategies, enhancing amenability for point‐of‐care applications.

While we have demonstrated clinically‐relevant sensitivity, to translate this promising platform toward clinical applications, it is pertinent to study the performance of our *Novabeads* under exposure to more complex biological samples that contain enzymes like nucleases and proteases. While this will be the focus of our future work, we have assessed the performance of *Novabeads* in simulated biological samples containing endogenous enzymes, specifically DNase I, prepared at expected serum concentrations for cancer patients–that is, 5 U mL^−1^.^[^ ^61^ ^]^ As shown in Figure S12 (Supporting Information), the standard beads (containing ssDNA‐based probes) resulted in a significant (≈47%) loss of signal in the presence of endogenous enzymes, owing to the enzyme‐driven degradation of the natural oligonucleotide probe within the hydrogel. In contrast, our *Novabeads* exhibited no detectable signal loss, owing to the resistance of our synthetic PNA probe to degradation by enzymes. This preliminary study demonstrates the amenability and practical utility of our platform under exposure to complex biological samples.

Finally, to assess the specificity of the *Novabead* biosensing platform, the FRET‐based analytical signal of the *Novabeads* was measured following the addition of a non‐target microRNA (miR‐141) and mutated sequences of the target miR‐16 containing single nucleotide polymorphisms (SNPs) at one or more locations along the sequence (oligonucleotide sequences shown in Figure 5D). As presented in Figure 5E, compared to the signal from the target miR‐16, which was normalized to 1, the non‐target sequences exhibited significantly lower fluorescence intensities, corresponding to roughly 20%, 12% and 1% of the target‐generated signal for miRNAs sequences with 1, 2, and 3 SNP mutations, respectively. The high specificity of the biosensing platform was primarily attributed to the bioreceptor design, herein, the PNA probe's inherent characteristics as well as the negatively charged amino acid residues.^[^ ^62^ ^]^ These results demonstrate the ability of the proposed strategy to discriminate between highly homologous miRNA sequences, even those differing by a single nucleotide.

## 3 Conclusion

We report herein the development and validation of smart, stimuli‐responsive, bioreceptor‐clicked, and graphically‐encoded hydrogel microparticles, termed *Novabeads*, which enable ultrasensitive, enzyme‐free, and fluorescence‐based detection of molecular biomarkers. The platform leverages stimuli‐responsive materials functionalized with synthetic nucleic acid bioreceptors based on PNA probes, to achieve enzymeless optical signal enhancement through three synergistic mechanisms: increased probe loading, enhanced target capture efficiency, and geometrical shrinkage‐driven signal amplification. These factors synergistically contribute to a remarkable ≈35 fold enhancement factor in the analytical signal. Following optimization of the *Novabead* platform, its analytical performance was characterized by implementing a proof‐of‐concept fluorogenic biosensing strategy for miR‐16 detection based on a FRET analytical signal. Compared to equivalent non‐responsive standard beads, *Novabeads* resulted in an outstanding one order of magnitude expansion in linear dynamic range and a 7.1‐fold improvement in LOD down to 28.8 pM (=2.9 fmol). Importantly, this fully materials‐driven approach offers equivalent or better analytical performance compared to other enzymeless approaches reported in the literature without the need for enzymes, nanomaterials, or thermal cycling. By rationally engineering smart hydrogel‐based materials for biosensing applications, this innovative approach introduces a new paradigm for next‐generation, ultrasensitive, and enzyme‐free biosensing platforms, offering a scalable, robust, and user‐friendly solution that can serve as a key enabler of precision diagnostics.

## 4 Experimental Section

### Materials

All reagents and solvents were from commercial suppliers and used without further purification. All oligonucleotides used in this study, including the single‐stranded DNA (ssDNA) probes and target miRNA, were purchased from Integrated DNA Technologies (IDT). Polyethylene glycol diacrylate (PEGDA, $M_{w}$ = 700), 2‐Hydroxy‐2‐methylpropiophenone (Darocur), sodium chloride (NaCl), and Tween‐20 were purchased from Sigma–Aldrich. Polyethylene glycol (PEG, Mw = 600) was purchased from Acros Organics. 100xTris‐EDTA (TE) buffer was purchased from EMD Millipore. Polydimethylsiloxane (PDMS, Sylgard 184) was purchased from Dow Chemicals. Nuclease‐free water was purchased from Omega Bio‐Tek. Fmoc‐Rink Amide MBHA Resin, Ethyl 2‐cyano‐2‐(hydroxyimino)acetate (Oxyma), Fmoc‐Asp (OtBu)‐OH, and Fmoc‐Cys(Trt)‐OH were obtained from Gyros Protein Technologies. Fmoc‐L‐Lys(N3)‐OH was obtained from VWR International. Fmoc/Bhoc‐protected PNA monomers were obtained from PolyOrg, Inc. Cy5‐NHS ester and Disulfo‐Cyanine3 DBCO were obtained from BroadPharm. Acetonitrile (ACN, Optima LC/MS and HPLC grades), Formic Acid (LC/MS grade), and *N*,*N*‐dimethylformamide (DMF, ACS reagent) were obtained from Thermo Fisher Scientific. *N*,*N*′‐diisopropylcarbodiimide (DIC), piperidine solution (20% in DMF), and triisopropylsilane (TIPS, 99%) were obtained from Sigma–Aldrich. Diethyl ether (HPLC grade) and trifluoroacetic acid (TFA, GPR grade) were obtained from VWR Chemicals. Dichloromethane (DCM, HPLC grade) was obtained from Honeywell Research Chemicals.

### PNA Probe Synthesis and Purification

All PNA sequences were synthesized at 0.02 mmol scale on Rink‐Amide MBHA resin (0.358 mmol g^−1^) using a PurePepChorus automated peptide synthesizer. Synthesis was carried out using a standard Fmoc solid‐phase peptide synthesis (SPPS) protocol, including systematically repeated steps of coupling and deprotection interspaced with washings (3 × 2 mL dimethylformamide (DMF)). Coupling was conducted as a double cycle using 4 equivalents of Fmoc‐PNA monomer (or Fmoc‐amino acid, where required) and DIC/Oxyma Pure as coupling reagents in DMF (4 mL) at 85 °C for 5 min. Deprotection was achieved by two cycles of 20% piperidine in DMF (3 mL) at 40 °C for 50 sec. PNA was cleaved from the resin using a cleavage cocktail of TFA/TIPS/H_2_O (95:2.5:2.5% v/v/v) at room temperature for 2.5 h. The resin was removed by filtration, and the filtrate was precipitated in ice‐cold diethyl ether. The precipitate was isolated by centrifuging on (4000 rpm, 10 min, 4 °C), dissolved in acetonitrile/water (70:30 v/v%), and then freeze‐dried. The crude PNA product was purified by semi‐preparative reversed‐phase high‐performance liquid chromatography on a Shimadzu LC‐20 HPLC system equipped with a C18 column (5 µm, 30 × 250 mm). Eluents used were 0.1% TFA in H2O (A) and 0.1% TFA in MeCN (B). Semi‐preparative runs were performed by applying a linear gradient (at 35 mL min^−1^) of 10% to 80% B over 30 min. Fractions collected during the HPLC purification were analyzed by reversed‐phase liquid chromatographic–electrospray ionization mass spectrometry (LC‐ESI‐MS) on a LC‐IDX Tribrid Orbitrap mass spectrometer equipped with C18 column (3.5 µm, 2.1 × 150 mm). Those analytical runs used 0–100% B gradient over 20 min at 0.5 mL min^−1^ as the eluents were 0.1% formic acid in H2O (A) and 0.1% formic acid in MeCN (B). Fractions found to contain exclusively the desired product (i.e., above 90%) were pooled and freeze‐dried. All PNA structures and LCMS validation are presented in the Supporting Information.

### PNA Cy5 Labelling

The N‐terminus capping of PNA with the NHS ester of Cyanine5 carboxylic acid (Cy5‐NHS ester) was carried out on resin in a syringe with a polypropylene frit (20 µm). After swelling for 30 min in DMF (3 mL), followed by solvent draining, the resin was mixed with a pre‐prepared solution of PBA‐NHS ester (4 eq.) and of N‐methylmorpholine (8 eq.) in DMF (3 mL), and allowed to react under gentle shaking overnight, at room temperature. The reaction was stopped by draining of the reaction mixture, and the resin was washed with DMF (3 × 5 mL), DCM (5 × 5 mL), and vacuum dried. Cy5‐labelled PNA was cleaved from the resin and purified by HPLC as described earlier.

### PNA Cy3 Labelling

DBCO‐Azide ligation in solution was employed herein, where PNA had an azide (N3) moiety and Cy3 had DBCO‐functionality. Accordingly, PNA was beforehand modified with an azide moiety by introducing a 6‐Azido‐L‐lysine residue at the N‐terminus during the SPPS step, and N3‐PNA was then cleaved from the resin and purified by HPLC using protocols described earlier.

DBCO‐Azide ligation was carried out off‐resin as follows: N3‐PNA (3.6 µmol) and Disulfo‐Cyanine3 DBCO (7.2 µmol) were mixed in 2 mL of acetonitrile/water (70:30 v/v%) to form a homogenous solution. The mixture was allowed to react under gentle shaking overnight, at room temperature. The product was isolated and purified by HPLC as described previously.

### Fabrication of Microfluidic Device for SFL‐Based Hydrogel Microparticle Synthesis

A microfluidic device for hydrogel particle synthesis was designed using SOLIDWORKS and fabricated via a cleanroom‐free approach combining 3D printing with hot embossing, as developed previously.^[^ ^63^ ^]^ Briefly, the design was printed with a Phrozen 3D printer (Phrozen Technology, Taiwan. The microfluidic channels were then transferred to a 1 mm‐PMMA sheet via hot embossing with a Sublym hot embossing machine (Eden Tech, Paris, France). Hot embossing was conducted at 400 MPa and 165 °C, heating was stopped after 15 min, while pressure was maintained until the temperature decreased to 90 °C to ensure complete feature replication onto the PMMA chip. The PMMA chip was subsequently used for PDMS molding. PDMS, mixed in a 10:1 (w/w) base‐to‐curing agent ratio, was cast onto the PMMA mold and cured at 60 °C for 2 h. The final devices were assembled and sealed by plasma bonding the PDMS on a PDMS‐coated glass cover slip (oxygen plasma treatment, 40 s), followed by annealing at 80 °C for 15 min to strengthen the bond.

### Synthesis of Hydrogel Microparticles via Stop‐Flow Lithography

Hydrogel microparticles were synthesized via stop flow lithography (SFL), which was reported previously. Briefly, a compressed air pressure source with an electrically powered valve was used to control the fluid (hydrogel precursor) flow inside the microfluidic channel. A shutter was also employed within an inverted fluorescence microscope to control the UV light exposure from a UV LED (Thorlabs M365L2) through a defined photomask for photopolymerization. An Arduino microprocessor was programmed to simultaneously control the fluidic valve and microscope shutter to enable the high‐throughput fabrication of hydrogel microparticles within the microfluidic channels. The hydrogel precursor solution, which was based on previous work and optimized for biosensing assays, consisted of 20% polyethylene glycol diacrylate 700 (PEGDA700), 40% polyethylene glycol 600 (PEG600), 35% 3xTris‐EDTA (3xTE) buffer, and 5% Darocur as a photoinitiator. For smart microparticles, the buffer was replaced with an equal volume of AA. During SFL, microparticles were formed by periodic UV exposure (300 ms) when the precursor was stopped inside the microfluidic channel. The synthesized microparticles were collected in a microtube (Eppendorf, US) filled with 100 µL of TET buffer (1xTE buffer and 0.05% Tween‐20). Following vortexing and centrifugation for 1 min, the microparticles were rinsed by removing 80 µL of the supernatant and resuspending in an equal volume of fresh TET buffer. The rinsing step was repeated 3 times. For smart microparticles, rinsing was repeated until the supernatant was the same as the fresh TET buffer (pH 8). Finally, the hydrogel microparticles were stored in 100 µL of TET at 4 °C until future use.

### Comparison of the Functionalization Efficiency Between Two Chemistries

For in situ functionalization (ISF), the hydrogel precursor solution was mixed with acrydite‐modified ssDNA‐Cy5 at a volume ratio of 9:1 such that the final concentration of ssDNA was 1 µM. Hydrogel microparticles were synthesized via SFL under identical UV light and exposure conditions as described previously. For the post‐synthesis functionalization (PSF) method, the hydrogel microparticles were synthesized via SFL under the same conditions. Thiolated PNA‐Cy3 was reduced in 0.5 mM of TCEP solution to achieve a PNA concentration of 1 µM. The blank hydrogel microparticles were then suspended in the reduced PNA solution to enable the thiol‐ene click reaction. The mixture was incubated in a thermoshaker at 37 °C with agitation at 1200 rpm for 120 h based on the optimization study shown in the (Figure S11, Supporting Information).

### Characterization of the Functionalization Factor (FF), Shrinkage Factor (SF), Capture Factor (CF), and Overall Amplification of the *Novabead* Microparticles

For characterizing FF, hydrogel microparticles were synthesized via SFL and post‐functionalized with the thiolated PNA‐Cy3. Following rinsing with rinse buffer (RB, TET buffer containing 50 mM NaCl), fluorescence images were measured using a fluorescence microscope at 20x magnification.

For SF characterization, hydrogel microparticles were suspended in an alkaline (pH 8) TE buffer. After rinsing, the microparticles were imaged with the fluorescence microscope, obtaining size and fluorescence intensity measurements. The microparticles were then transferred to 100 mM 2‐(N‐Morpholino)ethanesulfonic acid (MES) solution(pH 5) and incubated for 1 h. This pH‐triggered shrinkage step could be reduced from 1 h to less than 3 min, achieving the same result as shown in the Video S1 (Supporting Information). The microparticles were then imaged by a fluorescence microscope to characterize size and fluorescence intensity.

For characterizing CF and overall amplification, hydrogel microparticles were synthesized via SFL. 10 µM of PNA was used for biofunctionalization based on the described PSF method. Hybridization was performed with 100 nM miRNA‐Cy5 under room temperature (RT) with agitation at 800 rpm for 4 h. Images of the microparticles under alkaline and acidic conditions were captured under the fluorescence microscope. Microparticles were rinsed with RB three times between steps.

### Optimization To reduce Da‐Related Ring Effect

Hydrogel microparticles were synthesized via SFL with a 300 ms UV polymerization time. To optimize PNA concentration in the functionalized hydrogel microparticles, various concentrations of thiolated PNA (10 µM, 1 µM, 100 nM, and 10 nM) were used to functionalize hydrogel microparticles. The functionalized microparticles were hybridized with 100 nM miRNA‐Cy5, hybridized microparticles were imaged under the fluorescence microscope. To optimize polymerization to reduce the ring effect, hydrogel microparticles were synthesized via SFL, with varying UV exposure durations (300, 200, and 100 ms). Each duration was tested with two PNA concentrations (10 and 1 µM), and particles were hybridized with 100 nM miRNA‐Cy5. The microparticles (post‐miRNA hybridization) were then imaged under the fluorescence microscope.

### Implementing FRET‐Based Assay in Hydrogel Microparticles

Based on the optimized parameters for minimizing the Da‐related ring effect, hydrogel microparticles were synthesized via SFL, with 200 ms as the polymerization time. Post‐functionalization was carried out by incubating the microparticles with 1 µM thiolated 11‐mer‐Cy3 (TCEP reduced) under the same conditions previously described. Hybridization was performed with 100 mM miRNA and 1 µM 11‐mer‐Cy5 under RT with agitation of 800 rpm for 4 h. Microparticles were rinsed with RB three times between steps. Images of the hybridized microparticles under basic and acidic conditions were captured under the fluorescence microscope with the appropriate filter set for FRET (Semrock λ_ex_ = 531/40 nm, λ_em_ = 676/29 nm).

### Fluorescence Microscopy for Image Acquisition

Microscopy images or micrographs of the hydrogel microparticles were captured with a fluorescence microscope (Zeiss Axio Observer) equipped with an LED excitation source (X‐Cite 120 LED) using a 20x objective lens and the appropriate filter set based on the dye. Images were analyzed using ImageJ software.

### ATR‐FTIR Analysis

Attenuated total reflectance Fourier‐transform infrared (ATR‐FTIR) spectroscopy was used to monitor PEGDA polymerization, pH‐responsiveness of *Novabeads*, and PNA‐functionalization of standard beads through chemical composition analysis. Spectra were recorded on a Nicolet FTIR iS10 spectrometer equipped with Smart iTRTM Accessory (diamond crystal) and deuterated triglycine sulfate (DTGS) KBr detector using the associated OMNIC software. For controls, PEGDA and AA were added as a liquid, 1.5 µL, on the ATR crystal, while PNA was added as a powder. For hydrogel microparticles, suspensions of standard beads (in water without and with PNA in water) and *Novabeads* (in buffers MES at pH 5 and TET at pH 8) were added to the ATR crystal at 3 uL and left to air dry to eliminate the water signal. Background spectra of a clean ATR crystal in air were obtained before each sample. All spectra were recorded at an average of 256 scans with 4 cm^−1^ resolution between 4000 cm^−1^ and 525 cm^−1^ at room temperature.

## Conflict of Interest

The authors declare no conflict of interest.

## Supporting information



Supporting Information

Supplemental Video 1

## Data Availability

The data that support the findings of this study are available from the corresponding author upon reasonable request.
