# Supplementary material for: Novabeads: Stimuli‐Responsive Signal‐Amplifying Hydrogel Microparticles for Enzymeless Fluorescence‐Based Detection of microRNA Biomarkers
Source: Small. 2025 Jun 25;21(33):2503990. doi: 10.1002/smll.202503990 (PMC12372440; doi:10.1002/smll.202503990)
Supplement: Supplementary file 1 — Supporting Information [file SMLL-21-2503990-s001.docx]

Supporting Information

*Novabeads*: Stimuli-responsive signal-amplifying hydrogel microparticles for enzymeless fluorescence-based detection of microRNA biomarkers

Haoliang Lu, Fatimah Samman, Erol Hasan, and Dana Alsulaiman*

**Contents**

[**Successful Partial Polymerization of PEGDA: Standard Beads 2**](#_j4gri9kcgocg)

[**Incorporation of 35% AA into Standard Beads: pH-responsiveness of Novabeads 3**](#_jhisumoa1s90)

[**PNA Functionalization of Standard Beads 3**](#_l1rtg3bzmn0b)

[**Novabeads’ response to divalent (Ca2+) and trivalent (Fe3+) cations 5**](#_im32ezy7hqx7)

[**Comparison of this work to reported isothermal enzyme-free biosensors 6**](#_1vf4qahpxc8p)

[**Calibration Curve for Cy5 and Cy3 dyes 7**](#_tdiv4uftk7op)

[**LCMS validation of PNA identity 8**](#_v5tofuae7gz9)

[**Thiol-ene click reaction kinetics study 11**](#_bddp1m56z052)

[**Performance of Novabeads in simulated biological samples containing endogenous enzymes 11**](#_4r652ljc5ipp)

[**Biosensing Performance of Novabeads compared to Equivalent Solution-based Assay 12**](#_we07y89kbrc3)

[**References 13**](#_ugo94b5hujgi)

# **Successful Partial Polymerization of PEGDA: Standard Beads**


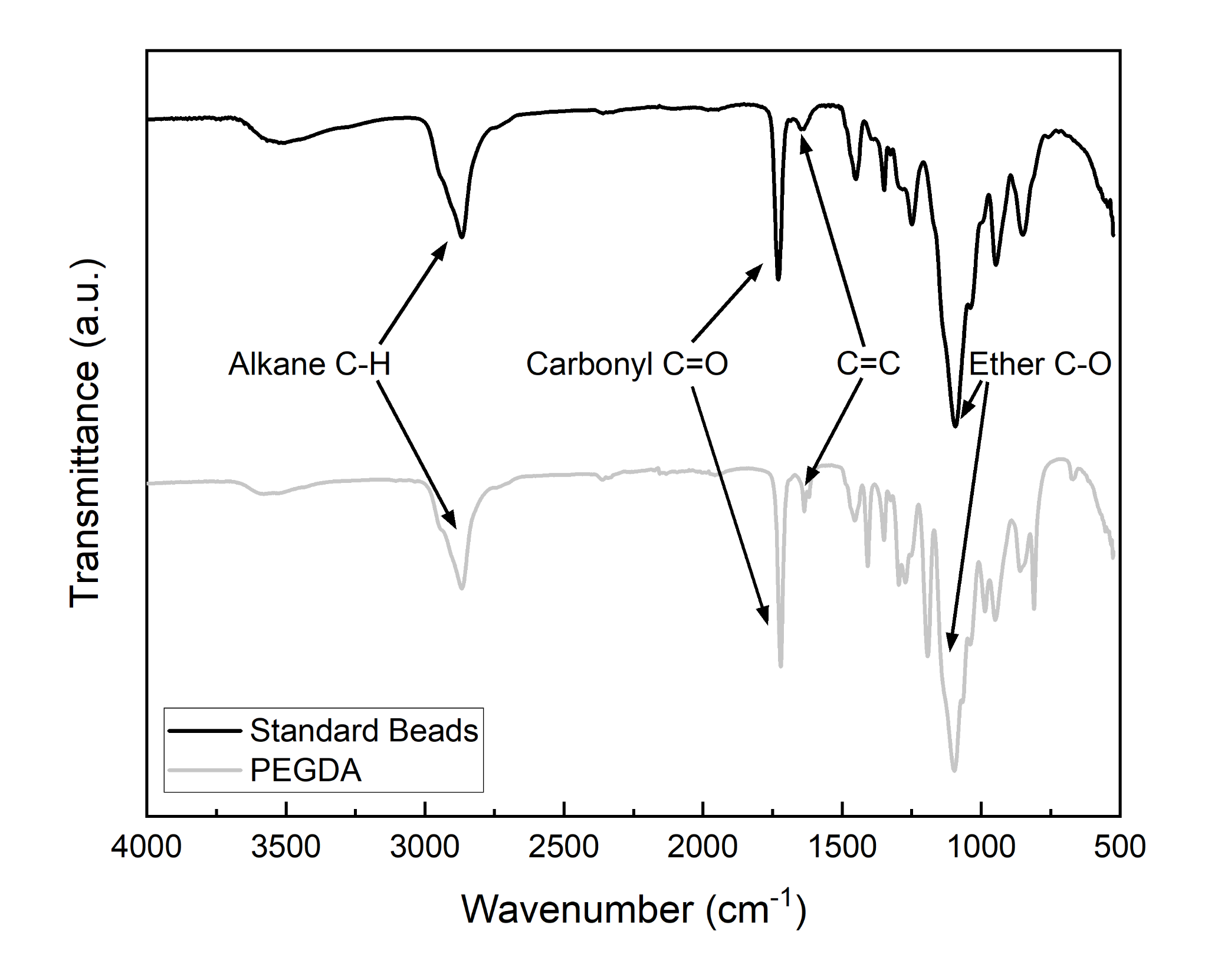


Figure S1. Full FTIR spectra of PEGDA before (gray) and after (black) radical photopolymerisation, showing a reduction in the C=C signal, which indicates the partial consumption of these bonds during polymerization. Detailed analysis can be found in **Figure 1C** of the main manuscript.

# **Incorporation of 35% AA into Standard Beads: pH-responsiveness of Novabeads**


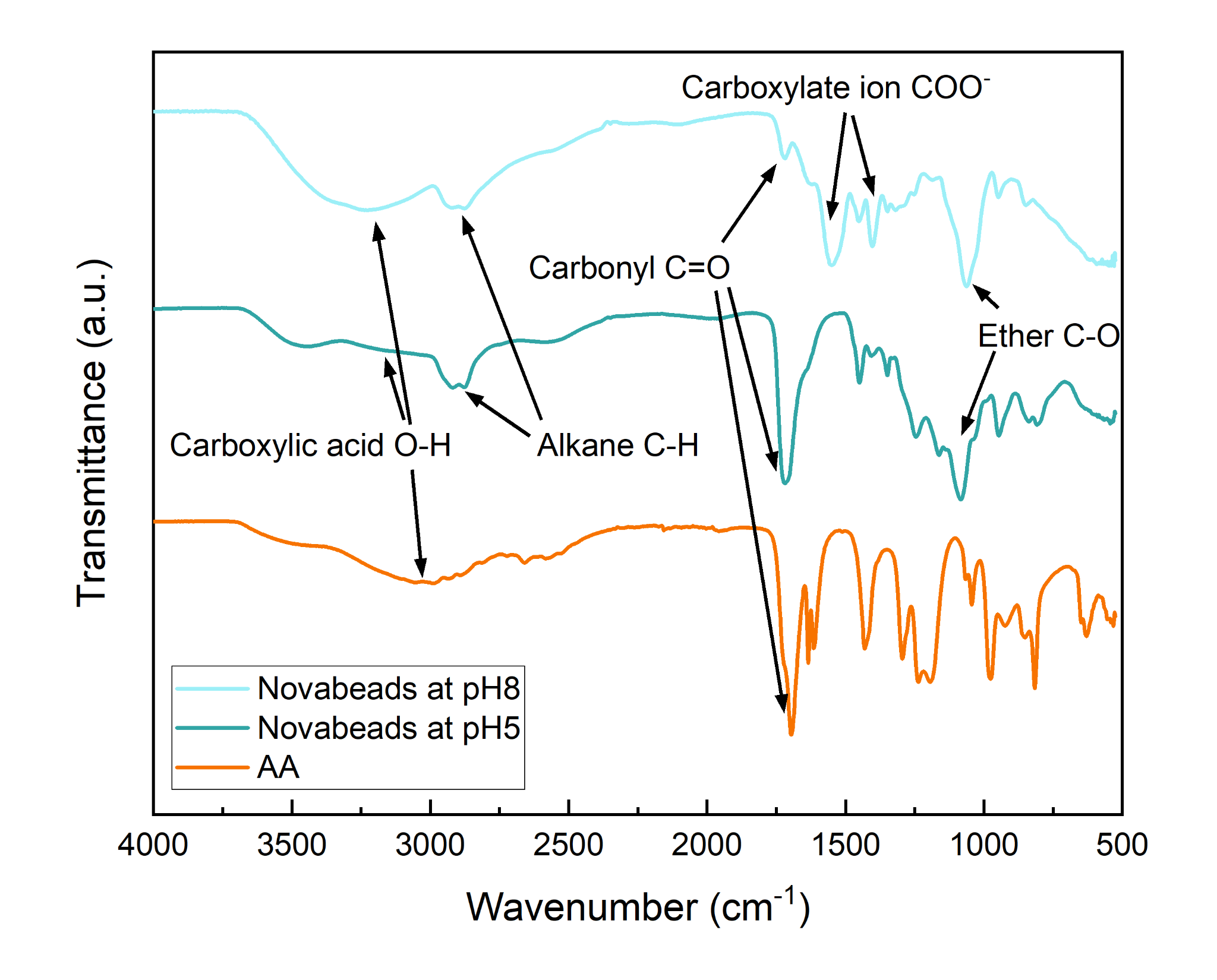


Figure S2. Full FTIR spectra of AA precursor (orange) and *Novabeads* in acidic (pH 5, light blue) and alkaline (pH 8, dark blue) conditions. For AA, a broad peak around 3040 cm^-1^ corresponding to the O-H stretch is observed. When AA is incorporated into the PEGDA hydrogel network, this peak is shifted slightly and its intensity changes depending on the pH, as the carboxylic acid group becomes either protonated or deprotonated. Detailed analysis can be found in **Figure 1C** of the main manuscript.

# **PNA Functionalization of Standard Beads**

To confirm successful functionalization of PNA, FTIR spectroscopy was employed to monitor specific spectral features. Unreacted C=C bonds in the standard beads are expected to react with S-H groups in thiolated PNA, leading to a decrease in C=C bond signal in the FTIR spectrum. Moreover, the PNA backbone contains amide bonds, which exhibit characteristic peaks around 1650 cm^-1^ (C=O stretch) and 1540 cm^-1^ (N-H deformation).[^[1,2]^](https://paperpile.com/c/WdRIDn/SbFwW+cxcPz)

The FTIR spectrum of PNA-functionalized standard beads was normalized to the C=O peak of the standard beads, while that of PNA remained unchanged to assist in peak assignment. We confirmed successful PNA functionalization, as evidenced by an increased amide peak intensity near 1650 cm^-1^ and the emergence of a new small peak around 1540 cm^-1^, compared to non-functionalized standard beads. Stretch vibrations of C-S and C-S-H were not observed, likely due to their weak absorption in the IR spectrue.[^[3]^](https://paperpile.com/c/WdRIDn/ZlNRl) The integrity of the standard beads' structure post-functionalization was verified by the retention of characteristic peaks, including the C-O and C-H stretches of the backbone[^[4]^](https://paperpile.com/c/WdRIDn/OP5Wy) and the C=O stretch from the carbonyl group,[^[4,5]^](https://paperpile.com/c/WdRIDn/OP5Wy+CnvVk) as previously discussed for PEGDA polymerization.


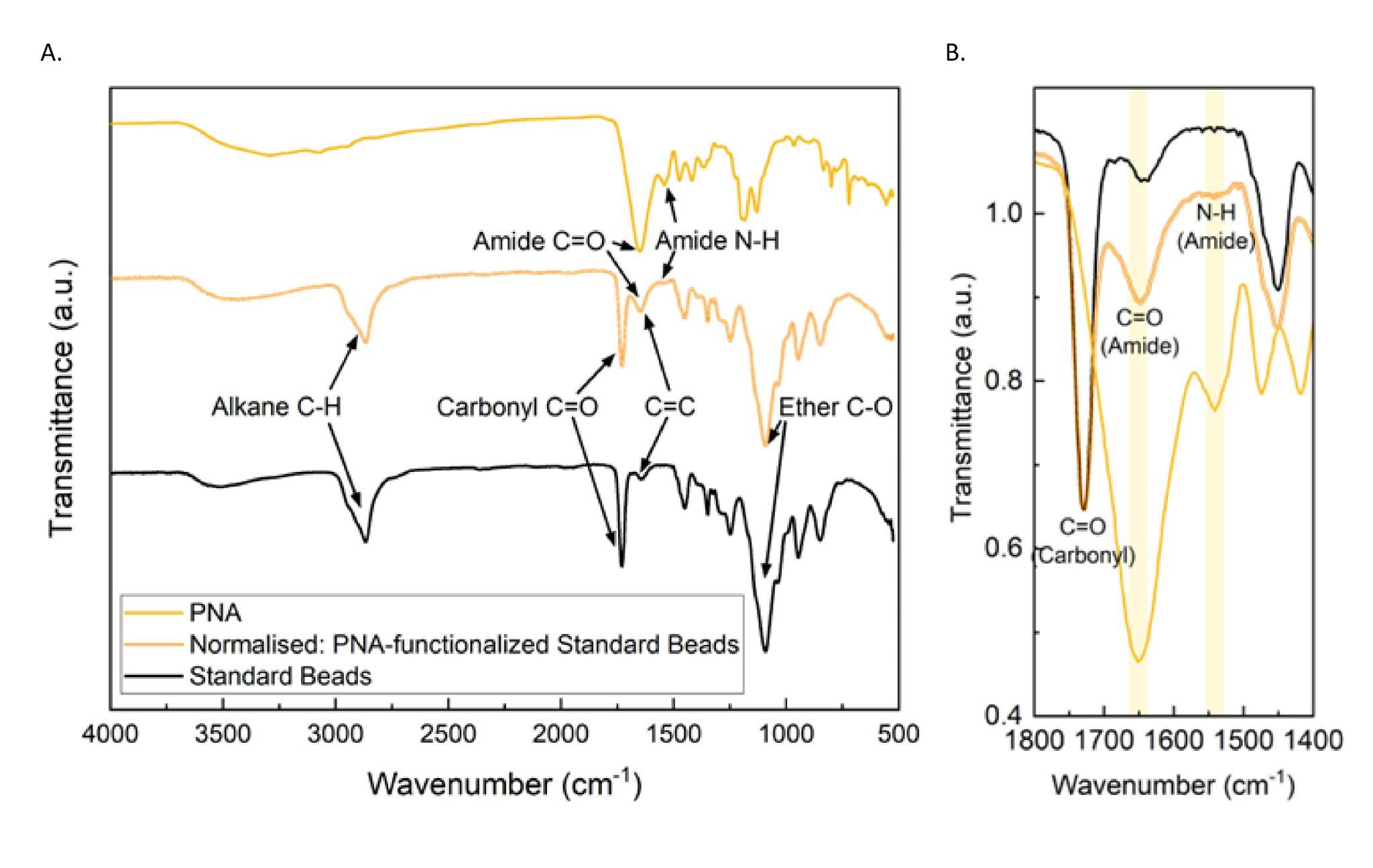


Figure S3. A. Full FTIR spectra of PNA (yellow), standard beads (black) and PNA-functionalized standard beads (orange). B. Zoomed FITR spectra showing amide bonds in PNA-functionalized standard beads. All transmittance values were converted from percentage to decimal for convenience (single line), and the PNA-functionalized standard beads spectrum was normalised (double line) to the C=O peak of the standard beads.

# ***Novabeads’* response to divalent (Ca^2+^) and trivalent (Fe^3+^) cations**


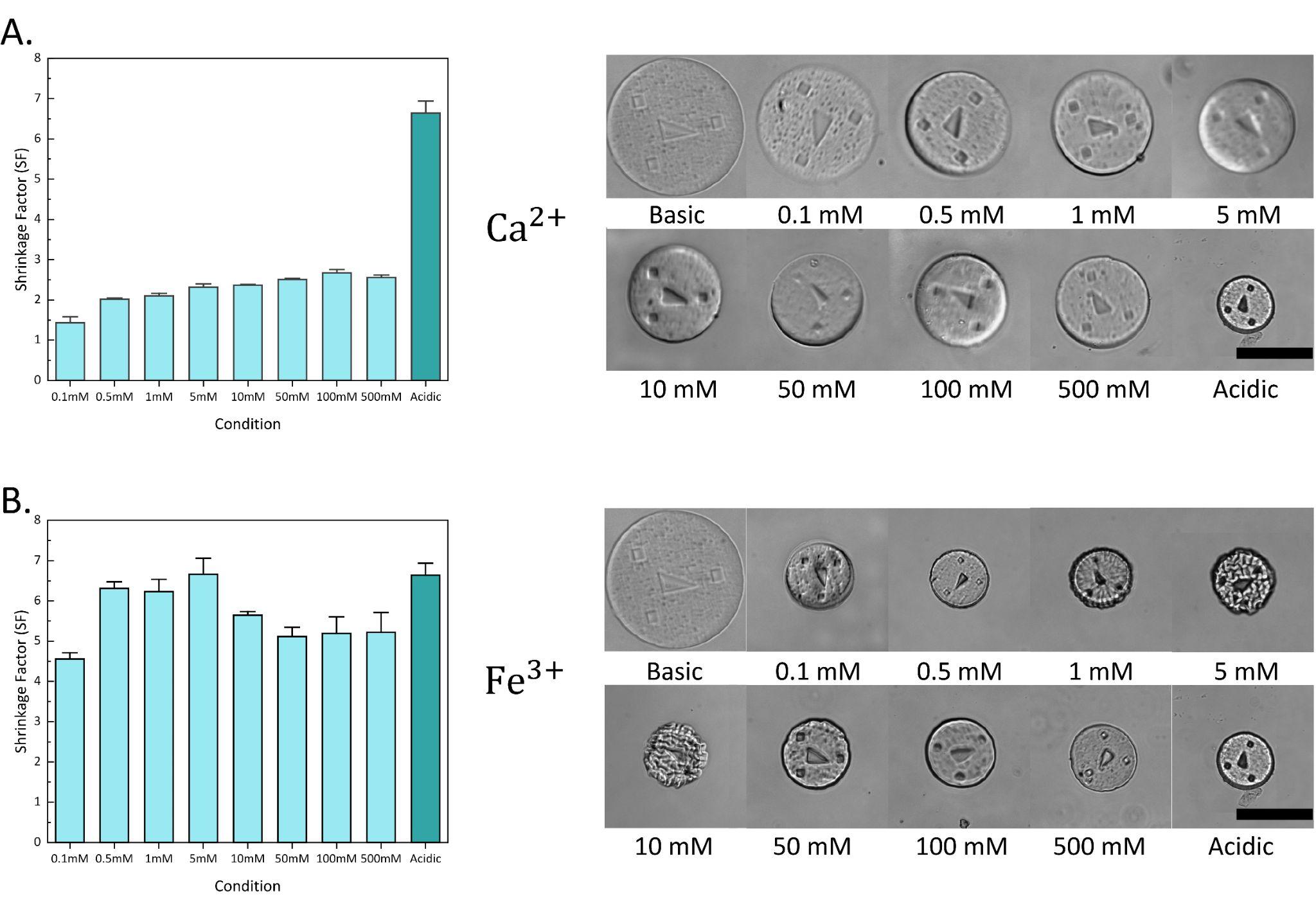


Figure S4. A. Calculated shrinkage factor and corresponding images of 35% Novabeads suspended in various calcium ion concentrations. B. Calculated shrinkage factor and the representative images of 35% Novabeads suspended in various ferric ion concentrations, showing surface disruption and morphological changes. The microparticle labelled “Basic” in panels A and B indicate the same representative Novabead under standard alkaline pH conditions (i.e., control condition with 0mM Ca^2+^ and 0 mM Fe^3+^). Similarly, the microparticle labelled “Acidic” in panels A and B indicate the same representative Novabead under standard acidic pH conditions (i.e., control condition with 0mM Ca^2+^ and 0 mM Fe^3+^).

This phenomenon was attributed to the strong crosslinking effect of ferric ions which leads to over-contraction and structural destruction of the hydrogel morphology. At low concentrations of cations, the negatively charged groups within the hydrogel network are neutralized, reducing the electrostatic repulsion and facilitating shrinkage. However, at higher cation concentrations, excessive monobindings between multivalent cations and carboxyl groups limit further shrinkage.[^[6]^](https://paperpile.com/c/WdRIDn/p25fs)

# **Comparison of this work to reported isothermal enzyme-free biosensors**

| Technique | | Materials | Dynamicrange | Limit of detection | Design complexity | Amplification steps | Multiplexed detection capability | Ref |
| --- | --- | --- | --- | --- | --- | --- | --- | --- |
| Nanomaterials-based | Photoinduced electron transfer | Cu nanoparticles | 0.5 to 100 nM  (>2 orders) | 0.2 nM | Moderate | 0 | No | [^[7]^](https://paperpile.com/c/WdRIDn/CVQ6j) |
|  | Light harvesting fluorescent probes | Polymeric nanoparticles |  | 1.3 pM | Moderate | 0 | No | [^[8]^](https://paperpile.com/c/WdRIDn/KwXXO) |
|  | Hairpin DNA-stabilized nanoclusters | Silver nanoclusters | 200 pM to 20 nM  (3 orders) | 200 pM | High | 1 | No | [^[9]^](https://paperpile.com/c/WdRIDn/5beWX) |
|  | Magnetic beads and GO-assisted enzyme-free signal amplification fluorescent biosensors | Magnetic beads and graphene oxide | 0 to 500 nM | 15.015 pM | Moderate | 1 | No | [^[10]^](https://paperpile.com/c/WdRIDn/0JG1G) |
|  | Quantum dots aggregation | Quantum dots | 20 pM to 100 pM(<1 order) | 14 pM | Moderate | 0 | No | [^[11]^](https://paperpile.com/c/WdRIDn/8rhyw) |
| Nanomaterials-free | Strand displacement reaction combined with hybridization chain reaction |  | 25 pM to 250 nM(4 orders) | 9.01 pM | High | 2 | No | [^[12]^](https://paperpile.com/c/WdRIDn/8pgaK) |
|  | Intercalator based fluorescence biosensor | Streptavidin magnetic beads | 113 nM to 2000 nM(>1 order) | 113.8 nM | Low | 0 | No | [^[13]^](https://paperpile.com/c/WdRIDn/ySUQc) |
|  | Catalytic hairpin assembly and entropy-driven amplification |  | 50 fM to 1 pM(>1 order) | 1.3 fM | High | 2 | No | [^[14]^](https://paperpile.com/c/WdRIDn/Azlj2) |
|  | ***Novabeads*** |  | **25 pM to 1000 pM(2 orders)** | **28.8 pM** | **Low** | **1** | **Yes** | **This work** |

Table S1. Comparison of this work with other enzyme-based fluorescence-based assays for miRNA detections in terms of sensitivity (limit of detection), design complexity (probes, primers,, etc), steps of amplification, and multiplexing capability.

Nanomaterial-based biosensing strategies can achieve pM limits of detection (LOD) with improved linear ranges. However, the integration of nanomaterials often introduces increased complexity and cost to the biosensing platforms. While nanomaterial-free approaches have demonstrated the capability to reach pM and even fM LODs, these methods typically require the design of multiple probes to amplify signals, which may compromise the robustness of the biosensing system and limit their capability for multiplexing.

# **Calibration Curve for Cy5 and Cy3 dyes**

Dye was introduced into microfluidic channels of the same height as the SFL microfluidic chips. Fluorescence intensity was recorded for the dyes prepared at varying concentrations.

**
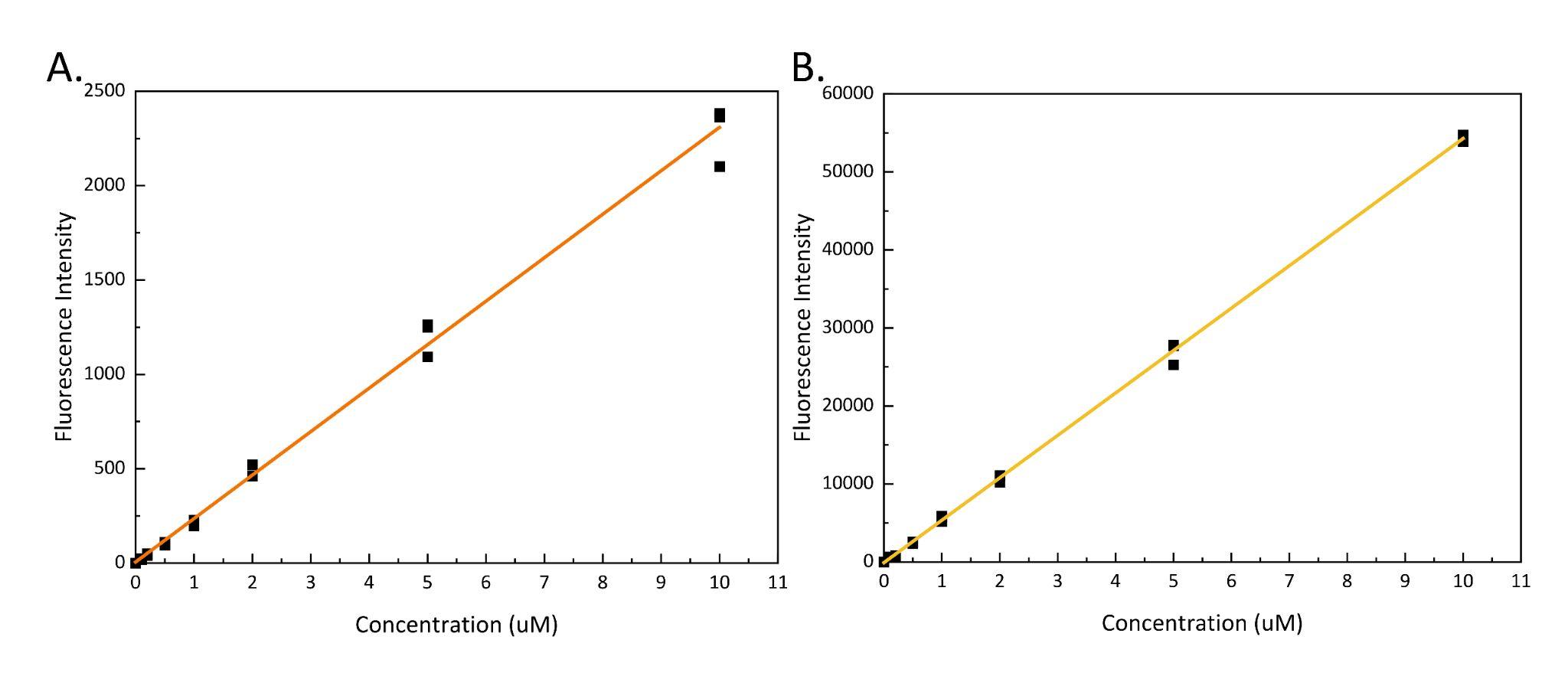
**

Figure S5. Calibration curves for Cy5 (A) and Cy3 (B).

# **LCMS validation of PNA identity**

PNA identity and purity was confirmed by comparing m/z (ESI+) observed to calculated values. ESI-MS data are reported as m/z observed (assignment = m/z calculated).

17mer-thiol-PNA: ((C to N), Cys-Asp-Asp-ATCGTCGTGCATTTATA)): 1236.48 ([4,941.75 + 4H]4+ = 1,236.44), 989.39 ([4,941.75 + 5H]5+=989.35), 824.65 ([4,941.75 + 6H]6+=824.62), 706.99 ([4,941.75 + 7H]7+=706.96), 618,74 ([4,941.75 +8]8+=618.72.


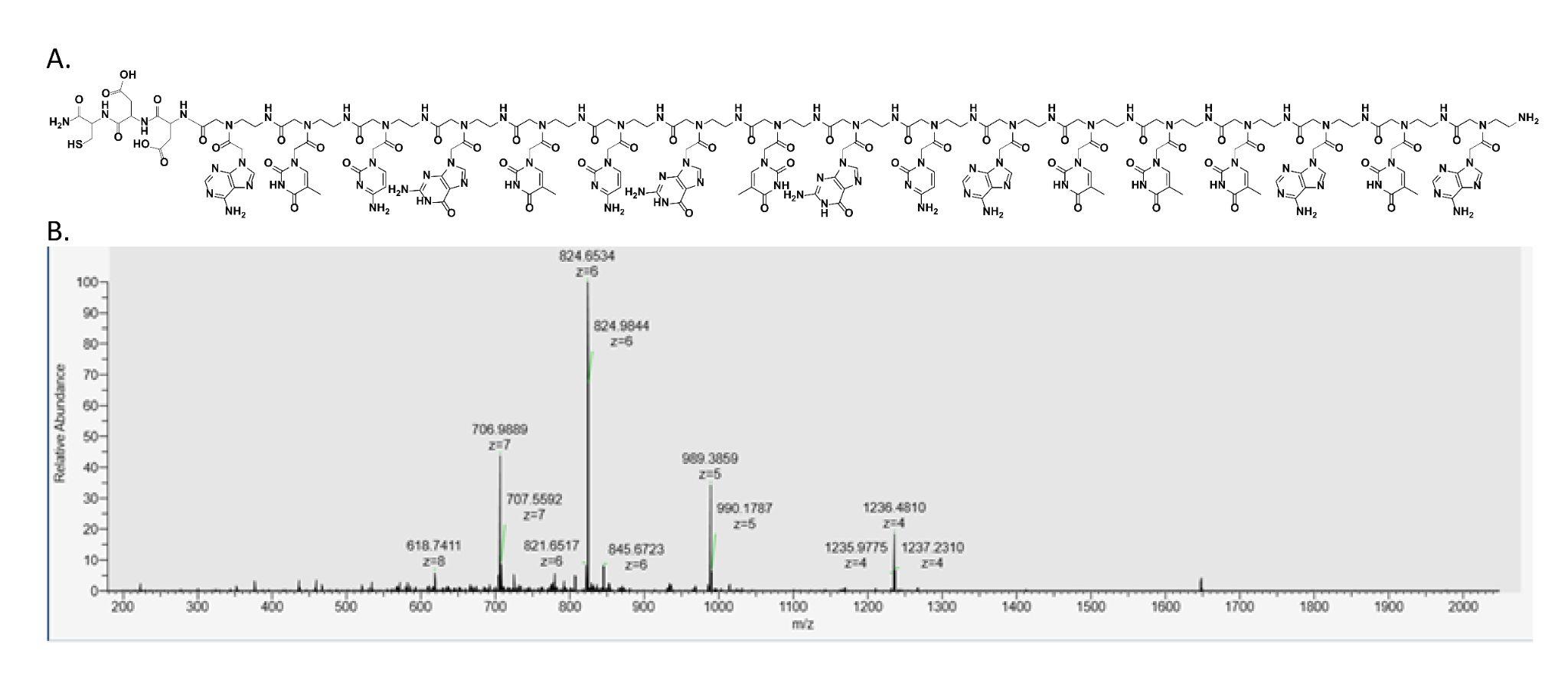


Figure S6. Chemical structure (A) and LCMS spectrum (B) of 17mer-thiol-PNA.

17mer-thiol-PNA-Cy3: ((C to N), Cys-Asp-Asp-ATCGTCGTGCATTTATA-Cy3)): 1346.29 ([5,382.44 + 4H]4+=1,346.61), 1077.24 ([5,382.44 + 5H]5+=1,346.61), 897.86 ([5,382.44 + 6H]6+=898.07), 769.74 ([5,382.44 + 7H]7+=796.74), 673.65 ([5,382.44 + 8H]8+=673.81), 598.91 ([5,382.44 + 9H]9+=599.04).


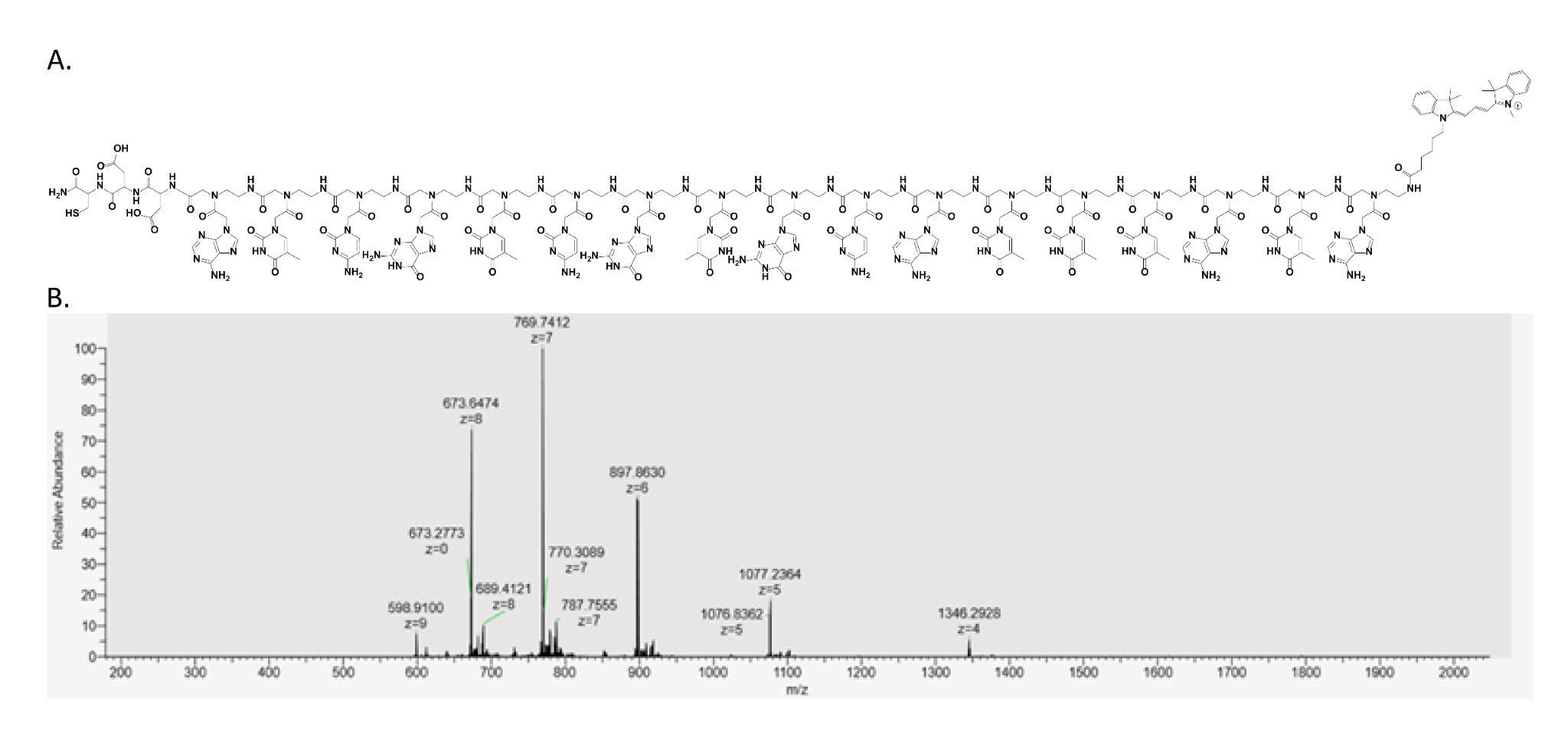


Figure S7. Chemical structure (A) and LCMS spectrum (B) of 17mer-thiol-PNA-Cy3.

Detector Probe: ((C to N), TTTATAACCGC-Asp-Asp-Cy5): 1216.86 ([3,648.63 + 3H]3+=1,217.21), 912.89 ([3,648.63 + 4H]4+=913.15), 730.52 ([3,648.63 + 5H]5+=730.72), 608.93 ([3,648.63 + 6H]6+=609.11), 522.08 ([3,648.63 + 7H]7+=522.23.


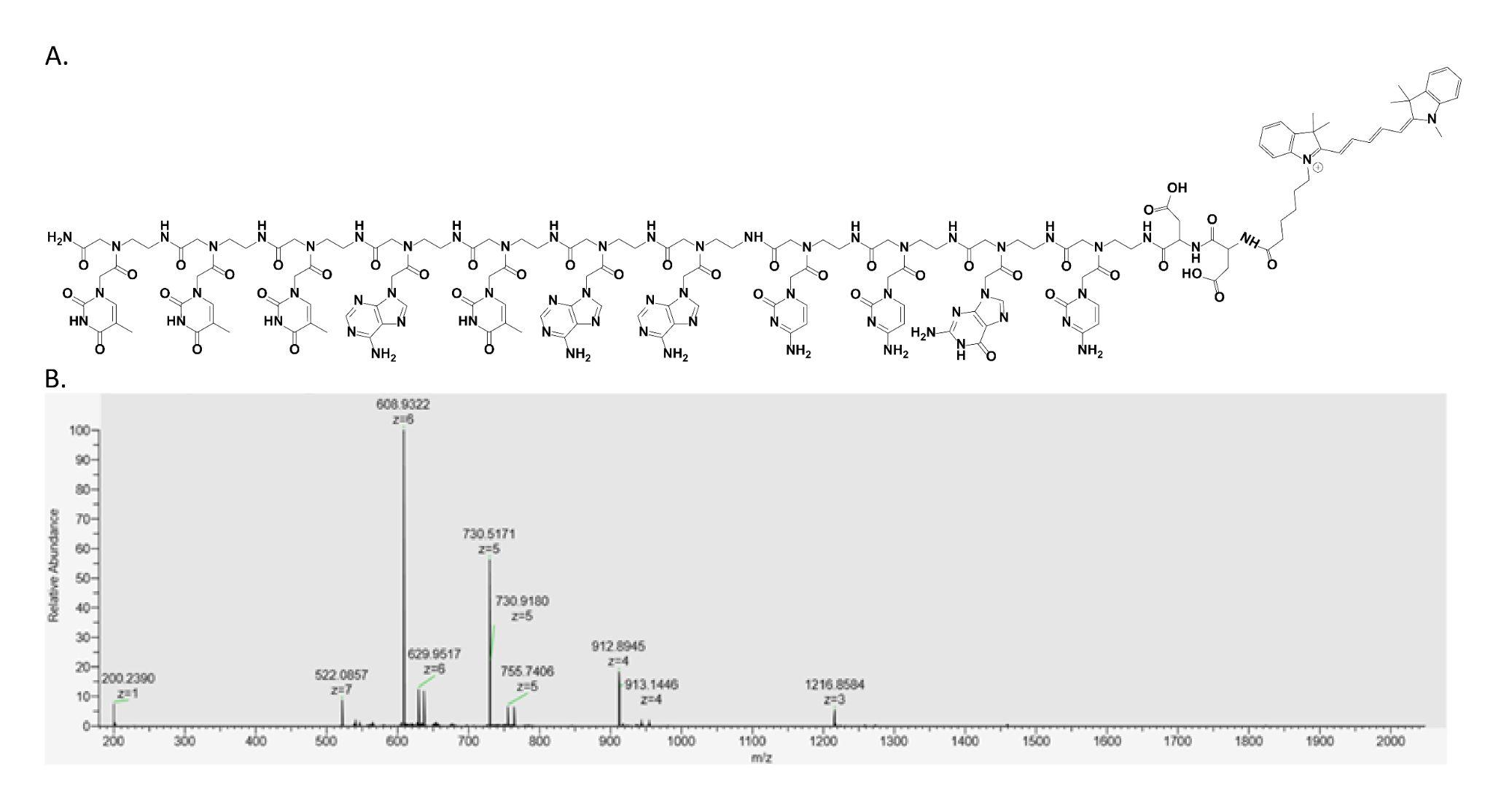


Figure S8. Chemical structure (A) and LCMS spectrum (B) of Detector Probe.

N3-PNA: (C to N), Cys-Asp-Asp-ATCGTCGTGCA-Lys(N3)): 1161.13 ([3,481.3 + 3H]3+=1,161.4), 871.10 ([3,481.3 + 4H]4+=871.32), 697.08 ([3,481.3 + 5H]5+=697.26), 581.07 ([3,481.3 + 6H]6+=581.21).


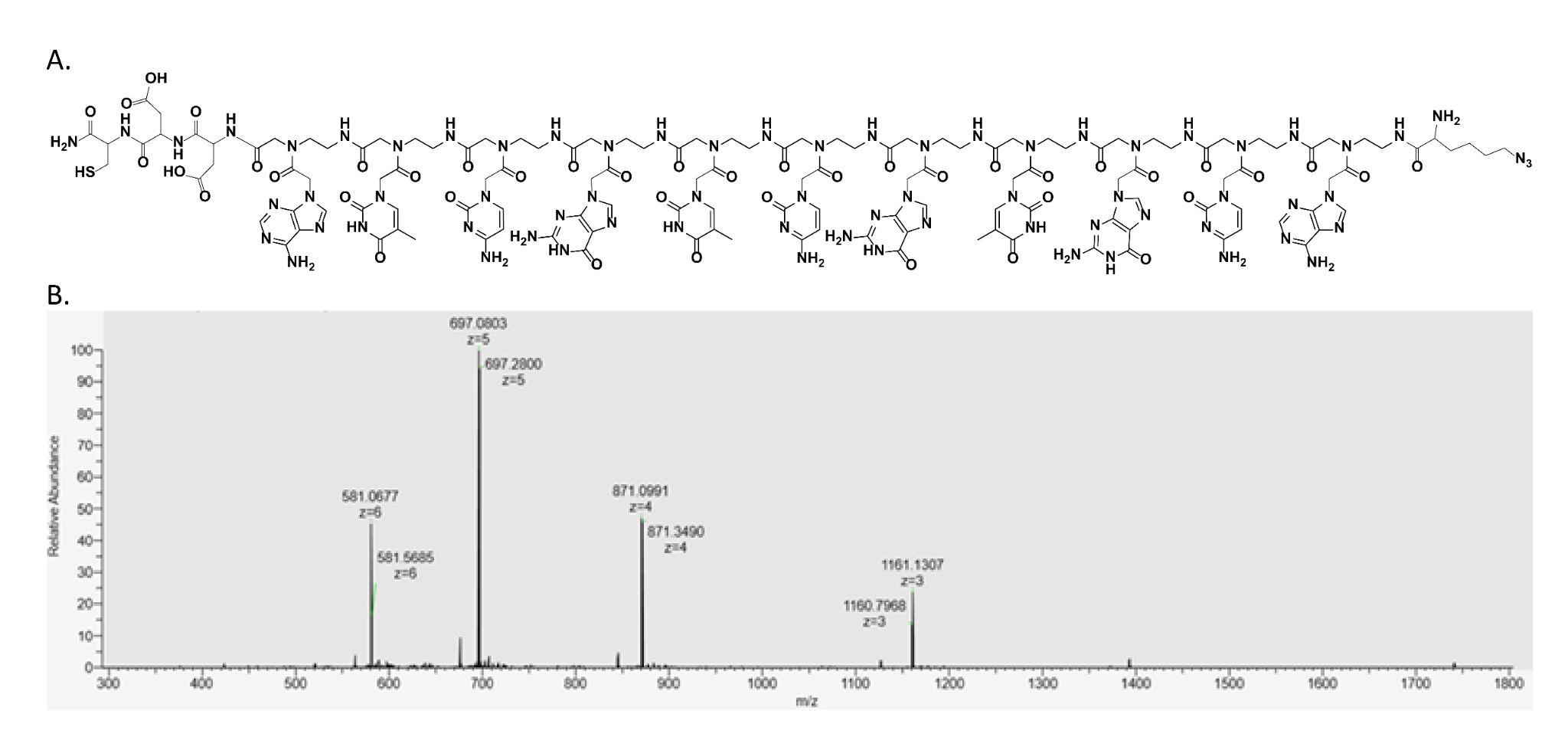
Figure S9. Chemical structure (A) and LCMS spectrum (B) of N3-PNA.

Capture Probe: ((C to N), Cys-Asp-Asp-ATCGTCGTGCA-Lys(triazole-DBCO- Disulfo-Cy3)): 1466.92 ([4,397.5 + 3H]3+=1,466.83), 1100.44 ([4,397.5 + 4H]4+=1,100.38), 880.55 ([4,397.5 + 5H]5+=880.50), 733.96 ([4,397.5 + 6H]6+=733.91).


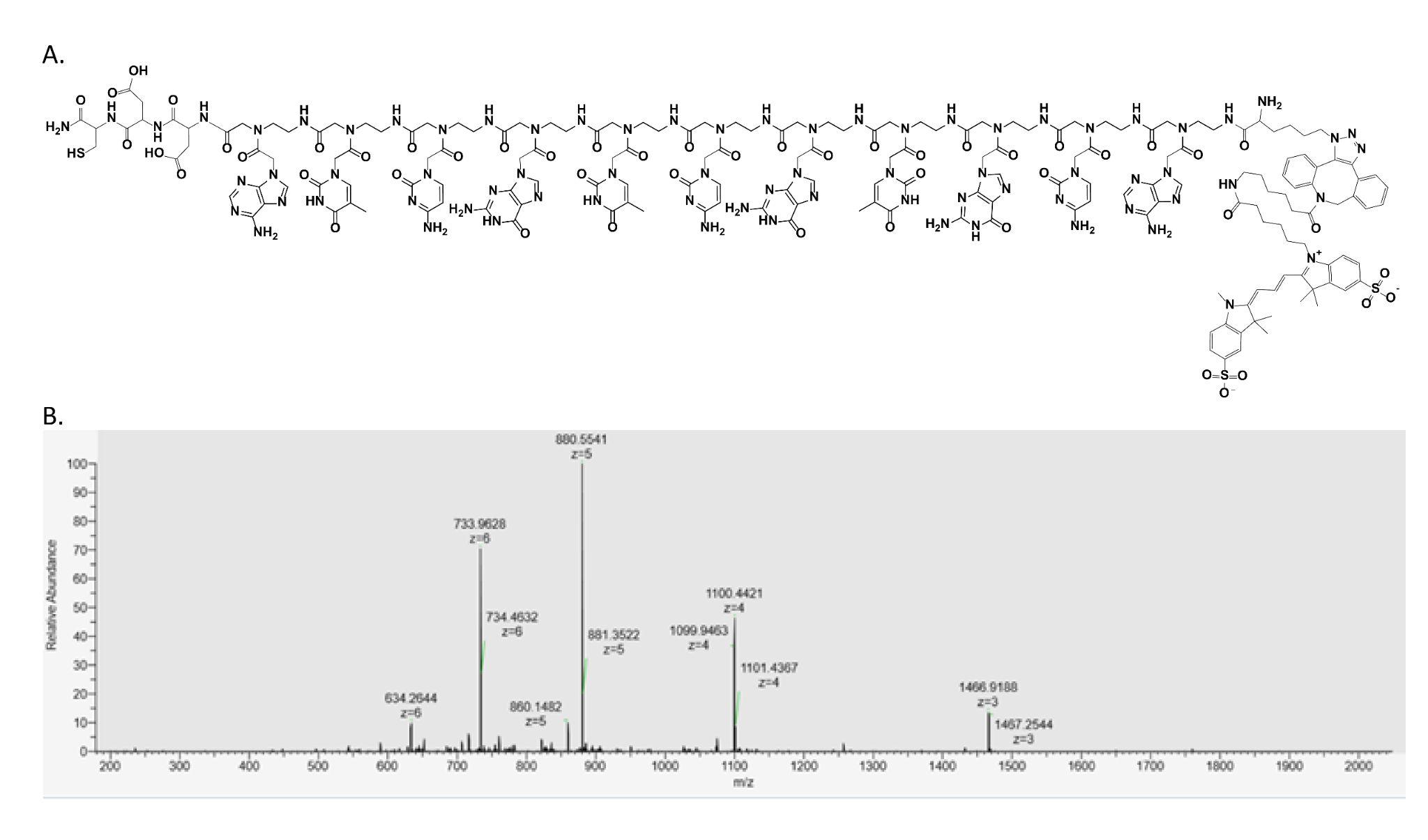


Figure S10. Chemical structure (A) and LCMS spectrum (B) of Capture Probe.

# **Thiol-ene click reaction kinetics study**

A kinetics study was conducted to investigate the thiol-ene click chemistry reaction and to optimize the incubation time between the thiolated probe and hydrogel microparticles. Specifically, we functionalized a thiolated PNA probe with Cy3 dye. The probe (prepared at 1 µM in TET buffer) was then incubated with the blank Novabead hydrogel microparticles, and the fluorescence of the microparticles was monitored over time until a threshold signal was reached. As shown in **Figure S11**, we observed that the reaction reached a threshold at roughly 120 hours; thus, this duration was chosen as the optimal condition for our future studies.


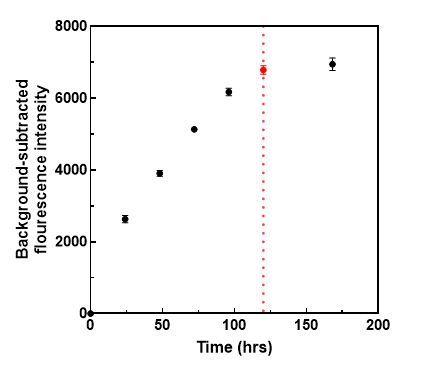


Figure S11. Kinetics of the thiol-ene reaction according to the reaction time. The reaction reached a threshold around 120 h from reaction initiation.

# **Performance of Novabeads in simulated biological samples containing endogenous enzymes**

This study assesses the performance of Novabeads in simulated biological samples containing endogenous enzymes, specifically DNase I, prepared at expected serum concentrations for cancer patients - that is 5 U/mL.^[[15]](https://paperpile.com/c/WdRIDn/d2guk)^ Two types of hydrogel microparticles (ssDNA-functionalized standard microparticles versus PNA-functionalized Novabeads) were first incubated in the standard hybridization buffer (grey bar, ‘-DNase I’) or buffer with DNase I (blue bar, ‘+DNase I’) overnight (~8h). Next, we measured the response to 100 nM of Cy5-labelled microRNA. The ssDNA probe used was purchased from IDT with the following sequence (equivalent to PNA that is complementary to miR16): 5’-ATATTTACGTGCTGCTA-3’. As shown in **Figure S12**, there was no difference in PNA-functionalized Novabeads with or without DNase I treatment, indicating that our probe is resistant to degradation by physiologically relevant enzymes. In contrast, there was a significant reduction in signal within the ssDNA-functionalized standard hydrogel microparticles after exposure to DNase I, owing to the degradation of the ssDNA probe by the nucleoase enzyme prior to miRNA capture. **Figure S12C** shows the percentage change in signal upon exposure to DNase I, showing a statistically significant drop in signal (47%, p<0.0001) for the ssDNA-functionalized microparticles. This study highlights the robustness of our synthetic probe and the promise of our biosensing strategy in real clinical applications.


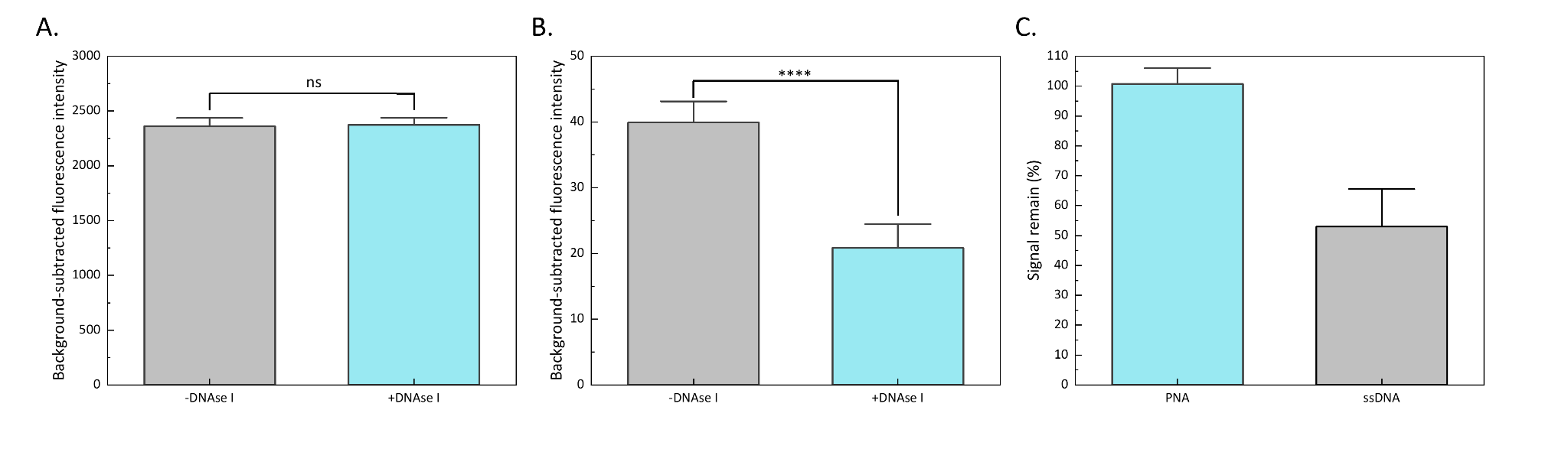


Figure S12. Change of fluorescence intensity of **A.** PNA-functionalized Novabeads (N>5) and **B.** ssDNA-functionalized standard beads (N>5) after incubation with DNase I. **C.** Signal change after DNase I incubation.

# **Biosensing Performance of Novabeads compared to Equivalent Solution-based Assay**


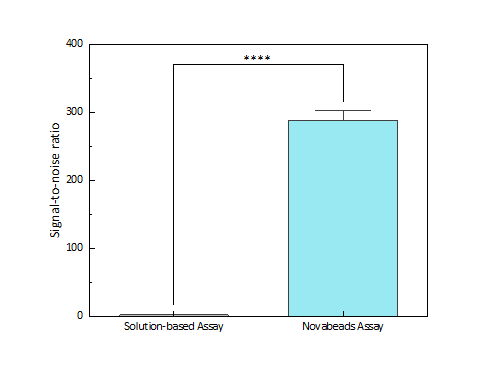


Figure S13. Comparison of miRNA sensing assay between solution and Novabeads in alkaline.

# **References**

[1] [E. Mateo-Martí, C. Briones, C. M. Pradier, J. A. Martín-Gago, *Biosens. Bioelectron.* **2007**, *22*, 1926.](http://paperpile.com/b/WdRIDn/SbFwW)

[2] [E. Mateo-Martí, C. Briones, E. Román, E. Briand, C. M. Pradier, J. A. Martín-Gago, *Langmuir* **2005**, *21*, 9510.](http://paperpile.com/b/WdRIDn/cxcPz)

[3] [Interpretation of Infrared Spectra, A Practical Approach, In *Encyclopedia of Analytical Chemistry*.](http://paperpile.com/b/WdRIDn/ZlNRl)

[4] [B. K. Pullagura, S. Amarapalli, V. Gundabala, *Colloids Surf. A Physicochem. Eng. Asp.* **2021**, *608*, 125586.](http://paperpile.com/b/WdRIDn/OP5Wy)

[5] [C.-H. Choi, J.-H. Jung, T.-S. Hwang, C.-S. Lee, *Macromol. Res.* **2009**, *17*, 163.](http://paperpile.com/b/WdRIDn/CnvVk)

[6] [J. Wang, L. Lou, J. Qiu, *J. Appl. Polym. Sci.* **2019**, *136*, 48182.](http://paperpile.com/b/WdRIDn/p25fs)

[7] [Q. Ma, S. F. Y. Li, *Microchem. J.* **2021**, *160*, 105646.](http://paperpile.com/b/WdRIDn/CVQ6j)

[8] [S. Egloff, N. Melnychuk, A. Reisch, S. Martin, A. S. Klymchenko, *Biosens. Bioelectron.* **2021**, *179*, 113084.](http://paperpile.com/b/WdRIDn/KwXXO)

[9] [J. Gu, Z. Qiao, X. He, Y. Yu, Y. Lei, J. Tang, H. Shi, D. He, K. Wang, *Analyst* **2020**, *145*, 5194.](http://paperpile.com/b/WdRIDn/5beWX)

[10] [J. Xu, M. Gui, H. Li, L. Nie, W. Zhao, S. Wang, R. Yu, *Anal. Chim. Acta* **2024**, *1306*, 342581.](http://paperpile.com/b/WdRIDn/0JG1G)

[11] [Y. S. Borghei, M. Hosseini, M. R. Ganjali, *J. Photochem. Photobiol. A Chem.* **2020**, *391*, 112351.](http://paperpile.com/b/WdRIDn/8rhyw)

[12] [J. Pu, M. Liu, H. Li, Z. Liao, W. Zhao, S. Wang, Y. Zhang, R. Yu, *Talanta* **2021**, *230*, 122158.](http://paperpile.com/b/WdRIDn/8pgaK)

[13] [P. Lavaee, S. M. Taghdisi, K. Abnous, N. M. Danesh, L. H. Khayyat, S. H. Jalalian, *Talanta* **2019**, *202*, 349.](http://paperpile.com/b/WdRIDn/ySUQc)

[14] [Q. Huang, K. Wang, Y. Wang, *Talanta* **2024**, *273*, 125928.](http://paperpile.com/b/WdRIDn/Azlj2)

[15] [R. M. Golonka, B. S. Yeoh, J. L. Petrick, S. J. Weinstein, D. Albanes, A. T. Gewirtz, K. A. McGlynn, M. Vijay-Kumar, *JNCI Cancer Spectr.* **2018**, *2*, ky083.](http://paperpile.com/b/WdRIDn/d2guk)
